# Multiple nanocages of a cyanophage small heat shock protein with icosahedral and octahedral symmetries

**DOI:** 10.1038/s41598-021-00172-2

**Published:** 2021-10-25

**Authors:** Sreeparna Biswas, Priyanka Garg, Somnath Dutta, Kaza Suguna

**Affiliations:** Molecular Biophysics Unit, Indian Institute of Science, Bangalore, 560 012 India

**Keywords:** Biophysics, Structural biology

## Abstract

The structures of a cyanophage small heat shock protein (sHSP) were determined as octahedrons of 24-mers and 48-mers and as icosahedrons of 60-mers. An N-terminal deletion construct of an 18 kDa sHSP of *Synechococcus sp.* phage S-ShM2 crystallized as a 24-mer and its structure was determined at a resolution of 7 Å. The negative stain electron microscopy (EM) images showed that the full-length protein is a mixture of a major population of larger and a minor population of smaller cage-like particles. Their structures have been determined by electron cryomicroscopy 3D image reconstruction at a resolution of 8 Å. The larger particles are 60-mers with icosahedral symmetry and the smaller ones are 48-mers with octahedral symmetry. These structures are the first of the viral/phage origin and the 60-mer is the largest and the first icosahedral assembly to be reported for sHSPs.

## Introduction

Small heat shock proteins (sHSPs) prevent the aggregation of cellular proteins during heat and other types of stress. They exist in dynamic equilibrium by the constant exchange of subunits between oligomers of various sizes. They have a broad substrate specificity and assemble in a variety of oligomeric forms. sHSPs are the smallest molecular chaperones with monomeric mass in the range of 12–40 kDa. They are unique as they are adenosine triphosphate (ATP)-independent. They are most ubiquitously present in all kingdoms of life and are upregulated during stress^1–3^. They play a vital role in maintaining protein homeostasis or proteostasis and are present in central nodes of the cellular proteostasis network. sHSPs are the front line of defence against the detrimental effects of cellular protein unfolding. They bind to a large variety of non-native proteins ranging from peptides to large proteins^4^ and efficiently prevent irreversible aggregation processes. They trap non-native proteins in a state from which they can be refolded to their native state with the assistance of ATP-dependent chaperones; thus, they act against protein aggregation^1,5^.

Each subunit of the dimeric building block of sHSPs contains a conserved α-crystalline domain (ACD) flanked by a variable N-terminal arm and a short C-terminal extension. The ACD has around 90 amino acids^6,7^ and is highly ordered. Despite their low sequence identity, the compact immunoglobulin-like structures of the ACDs of sHSPs are the most conserved across species. Crystal structures show that the sHSPs of metazoans and non-metazoans have different modes of dimerization^8,9^. The variation in the N- and C-termini affects the substrate interaction and oligomeric status of sHSPs^5^.

The N-termini were shown to play a major role in interacting with the substrates and the C-termini mediate dimer-dimer interactions leading to higher oligomer formation. The ability of sHSPs to assemble into multiple oligomeric species and bind to a large number of substrates indicates that they could exhibit diverse modes of substrate recognition and interaction. Studies using a variety of biochemical, biophysical and structural techniques provided insights into the mechanism of substrate binding and the necessity for the formation of different oligomers by sHSPs^4,10^. Upon heat and other types of stress, large oligomers were shown to dissociate into smaller ones in which the hydrophobic regions of the N-termini are exposed, enabling them to form complexes with denatured substrates. The complexes can then reassociate to form bigger oligomers again as observed in *Mycobacterium tuberculosis* HSP16.3^11^, *Escherichia Escherichia coli* IbpB^12^, pea HSP18.1^13^, wheat/*Triticum aestivum* HSP16.9^14^ and pea mitochondrial HSP22^15^. There are also a few instances where only active oligomers of smaller size (dimers/monomers/tetramers) exist as in Tsp36 of tapeworm^16^, *Arabidopsis thaliana* HSP18.5^17^ and human HSP27^18^. A single subunit of *Arabidopsis thaliana* HSP21 was also shown by cryo-EM analysis to partially unfold at the N-terminus and bind to the dimeric substrate, 1-deoxyD-xylulose 5-phosphate synthase^19^. The involvement of the N-terminus was also observed in other sHSPs. A conformational switch of the N-terminus to bind to substrates was revealed by crystallographic analysis of *Sulfolobus solfatricus* HSP14.1^20^. In pea HSP18.1, multiple substrates-binding sites on the N-terminus and a few on the ACD were identified^21^. Cryo-EM analysis revealed that T4 lysozyme is located inside the cage-like structures of the 24-mers and 48-mers of *Methanocaldococcus jannaschii *HSP16.5 interacting with the N-termini^22^.

**Figure 1:**
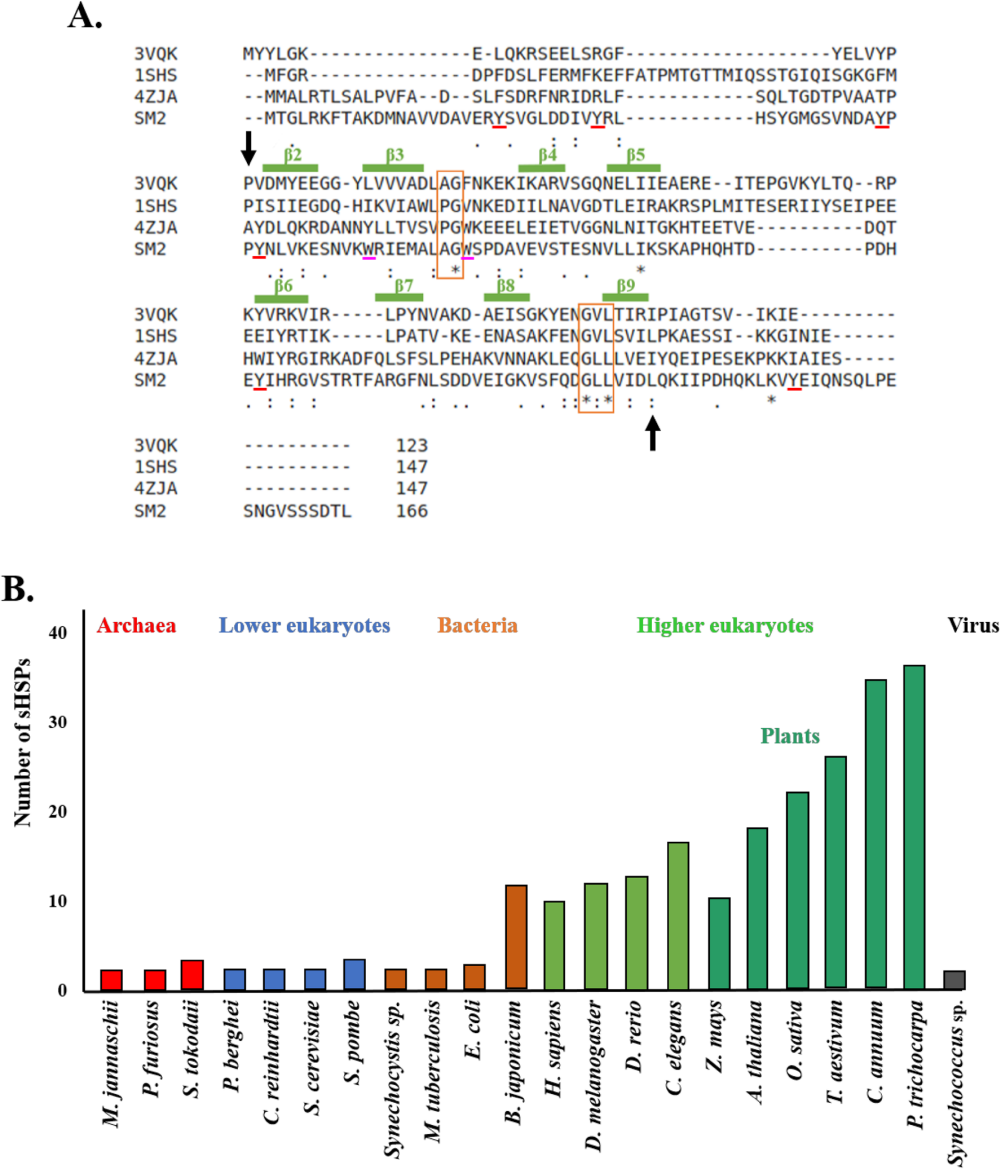
Sequences and occurrence of sHSPs (**A**) Structure-based sequence alignment of *Sulfolobus tokodaii* HSP14.0 (PDB code: 3VQK), *Methanocaldococcus jannaschii* HSP16.5 (PDB code: 1SHS) and *Salmonella* *typhymurium* AgsA (PDB code: 4ZJA) with the aligned SM2 sequence. The P-G and G-X-L motifs are shown in orange boxes. Arrowheads indicate the ACD. The secondary structural features shown in green correspond to *S. tokodaii* HSP14.0. The Tyr and Trp residues are highlighted. (**B**) Number of sHSPs shown in a representative set of different organisms.

sHSPs in archaea, bacteria, fungi, plants and animals have been reported earlier; nevertheless, the existence of viral sHSPs was not known until about 10 years ago. Sullivan and his colleagues^23^ were the first to detect the presence of sHSP genes in the marine viruses of *Synechococcus* and *Prochlorococcus* species. By phylogenetic analysis, Maaroufi and Tanguay^24^ found that cyanophage sHSPs form a monophyletic clade closer to bacterial class A sHSPs than to cyanobacterial sHSPs. The phage sHSPs contain a conserved ACD flanked by a relatively-conserved N-terminal arm and a short C-terminal extension with or without the L-X-I/V motif. The presence of a signature motif P-P-[YF]-N-[ILV]-[IV]-x(9)-[EQ] in the ACD region was reported in cyanophage sHSPs^24^. Cyanophage sHSPs have a characteristic A–G doublet, which is present in bacterial class A sHSPs^25,26^ (Fig. 1A). Most bacteria (with the exception of Rhizobia) and archaea typically have one or two sHSPs^27–31^ whereas higher organisms contain more sHSPs: 10 in humans, 12 in *Drosophila melanogaster* and 16 in *Caenorhabditis elegans*; in plants, the number is even higher (Fig. 1B)^1,32^. Plant sHSPs have the unique property of being associated with many organelles such as cytosol, nucleus, endoplasmic reticulum, mitochondria, chloroplast and peroxisome. All other sHSPs are cytosolic or nuclear with the exception of two mitochondrial sHSPs reported in *Drosophila melanogaster*^33^ and *Toxoplasma gondii*^34^. Of all the viruses known so far, only some strains of the cyanophages of two related species *Synechococcus* and *Prochlorococcus* have one sHSP each^24^.

sHSPs from the cyanophage S-ShM2 (HspSP-ShM2) and its bacterial host *Synechococcus sp.* WH7803 were characterized by Bourrelle-Langlois et al.^35^. HspSP-ShM2 showed chaperone activity on a few protein substrates during heat stress. It has a highly polydisperse profile and showed extensive subunit rearrangements and quaternary structural dynamics in solution. A large variation was observed in its oligomeric state with changing temperature and concentration.

Cyanophages are abundant and form an important component of fresh water and marine ecosystems as they regulate cyanobacterial colony compositions and recycle photosynthetically-produced organic carbon^36,37^. The exclusive presence of sHSPs in very few phages and their absence in other viruses hint at a possible unique role played by sHSPs in their survival under stress conditions. So far, the structures of viral sHSPs are not known and hence, we have taken up the already-characterized HspSP-ShM2^35^ for further study, with a special focus on the structural aspects, and in particular, to investigate how the 3D structures of viral sHSPs compare with the reported structures of their non-viral counterparts at different levels of organization. In order to probe the roles played by the termini in oligomerization and chaperone activity, and to establish a link between these two properties, we initiated structural and functional characterization of the full length and various truncation constructs of HspSP-ShM2 (SM2 in short). The chaperone activity of the constructs was investigated using chemically-denatured lysozyme. The size and content of the oligomers were determined by size exclusion chromatography in conjunction with multi angle light scattering (SEC–MALS) and dynamic light scattering (DLS). The importance of the C-terminal I-X-I motif in higher oligomer formation and the role of the N-terminus in the chaperone activity and oligomerization of sHSPs were examined. Crystallization of all the constructs was attempted; however, crystals of only one N-terminal deletion construct could be obtained and it diffracted to a resolution of 7 Å. The construct crystallized as a 24-mer with 432 symmetry. The dimeric association is similar to that found in the sHSPs of non-metazoans. As sHSPs tend to form large oligomers with extensive symmetry making them good candidates for electron microscopy (EM) experiments, we have used this technique as well to determine the structure of SM2. The negative stain EM images showed that the purified full-length protein is a mixture of particles having a major population of large cage-like assemblies and a minor population of smaller cages. The 3D structures of both have been determined by electron cryomicroscopy (cryo-EM) 3D image reconstruction at a resolution of 8 Å. The bigger particles are 60-mers with icosahedral symmetry and the smaller ones are 48-mers with octahedral symmetry. The crystallographic and EM studies show that SM2 is capable of forming various types of oligomers, which vary in size and symmetry. These are the first structures of viral or phage sHSPs to be reported. The 60-mer is the largest particle of the reported sHSP structures and the icosahedral or 532 symmetry has been observed for the first time in sHSPs. Despite variations, certain common features were observed in the three particles: the 24-, 48- and 60-mers. Sub-assemblies of hexamers made up of three dimers with 32 symmetry are present in all the three oligomers. Though the structures were determined at low resolutions, it is clear that the C-terminal I-X-I motif interacts with neighbouring molecules in a way similar to that observed in other sHSP structures. The flexible C-terminal tail changes its orientation relative to the ACD and hence, it enables the alternate packing of dimers in different oligomers. This study has revealed new features related to the oligomerization and symmetry of sHSPs.

## Results and discussion

### Multimer formation by the full length and deletion mutants of SM2

**Figure 2:**
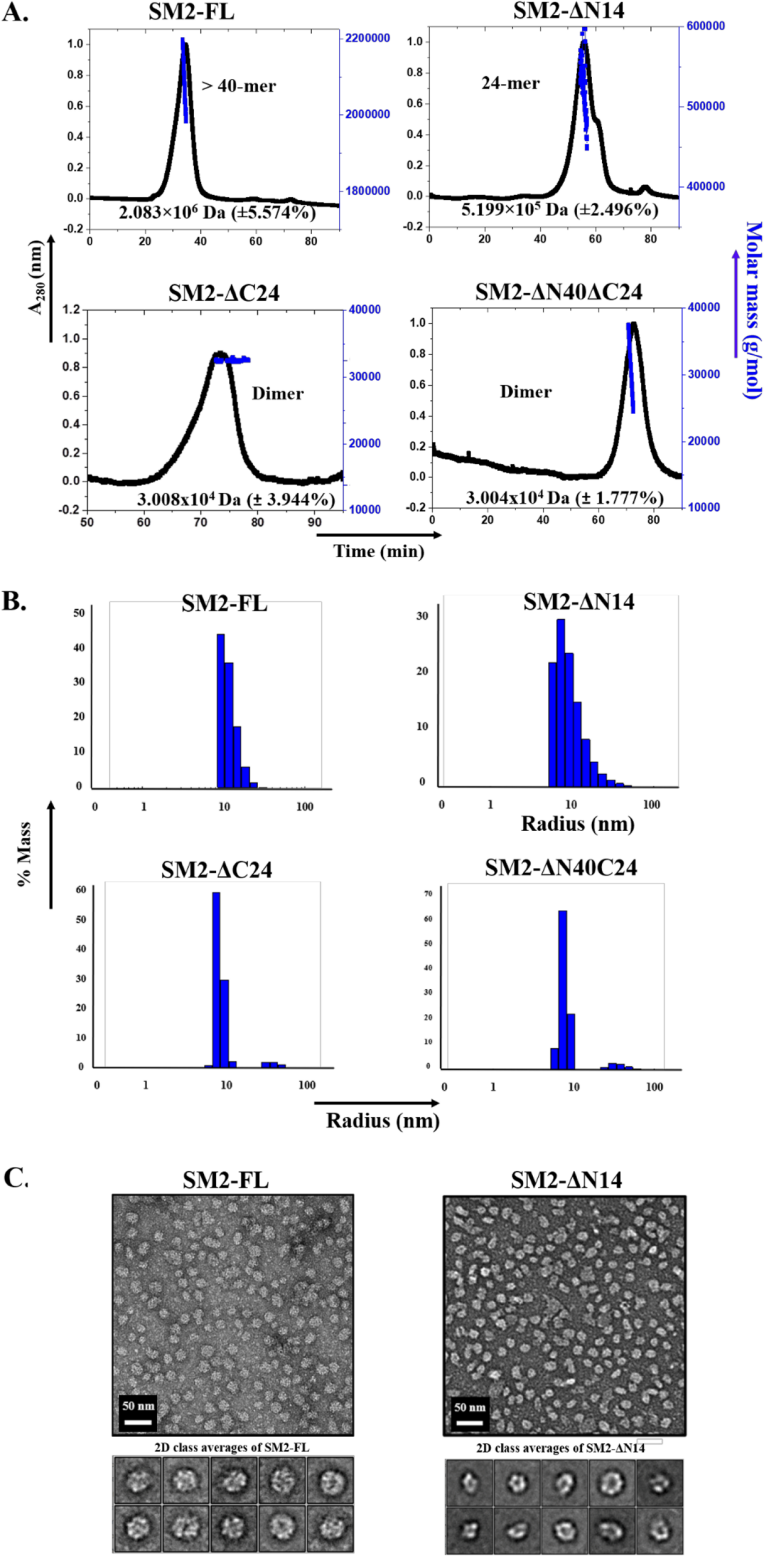
Characterization of SM2 oligomers (**A**) SEC-MALS and (**B**) DLS profiles of SM2 constructs: SM2-FL, SM2-ΔN14, SM2-ΔC24 and SM2-ΔN40ΔC24. (**C**) Negative stain micrographs of SM2-FL and SM2-ΔN14 with the 2D class averages.

We generated the full length (SM2-FL, 166 residues) and three deletion constructs: SM2-ΔN14, SM2-ΔC24 and SM2-ΔN40ΔC24 (Fig. S1A, Table 1) and purified (Fig. S1B) by affinity chromatography. The oligomer sizes were determined by SEC–MALS (Fig. 2A) and their polydispersity was measured by DLS (Fig. 2B). Circular dichroism (CD) of all the four constructs showed spectra that were characteristic of a β-sheet structure (Fig. S1C). However, the oligomeric status of the constructs, determined by SEC-MALS, showed a large variation. The constructs were polydisperse due to the presence of multiple oligomeric forms in solution. SM2-FL showed the formation of large oligomers (> 40-mers), where the mass corresponding to the peak apex was more than 900 kDa (the monomer mass was 21.03 kDa). The SM2-ΔN14 construct showed the formation of 24-mers in solution. The deletion of the L-X-V motif at the C-terminus resulted in the formation of dimers of the two constructs SM2-ΔC24 and SM2-ΔN40ΔC24. Thus, the C-terminal residues appear to play an important role in the formation of SM2 oligomers.

**Table 1:** Calculated molecular weight and theoretical pI of different SM2 constructs.

Construct	Mol wt (Da)	Theoretical pI
SM2-FL	21,031	6.20
SM2-ΔN14	19,596	5.96
SM2-ΔC24	18,412	6.33
SM2-ΔN40ΔC24	14,094	6.28

DLS experiments were performed on different constructs to check the polydispersity and to determine the hydrodynamic radii (Rh) in solution (Fig. 2B, Table S1). SM2-FL and SM2-ΔN14 had similar polydispersity profiles and Rh values (12 nm and 10.3 nm, respectively). The C-terminal deletion constructs without the L-X-V motif showed significantly smaller Rh values of 8.1 nm for SM2-ΔC24 and 7.7 nm for SM2-ΔN40ΔC24. The size appears to increase as the number of residues in the constructs increases.

As SM2-FL and SM2-ΔN14 form higher oligomers, these two constructs were screened by negative stain EM for cryo-EM studies. The negative stain EM showed a population of monodisperse particles with a diameter of ~ 25 nm for the SM2-FL construct while the SM2-ΔN14 construct was heterogeneous with the diameters of the particles ranging from 12.5 to 25 nm as shown in 2D class averages (Fig. 2C). The populations of SM2-FL oligomers were more homogeneous, non-aggregated, and of uniform size with large cage-like structures, suggesting that they are well-suited for the single particle analysis technique.

### The termini are required for the chaperone activity

**Figure 3:**
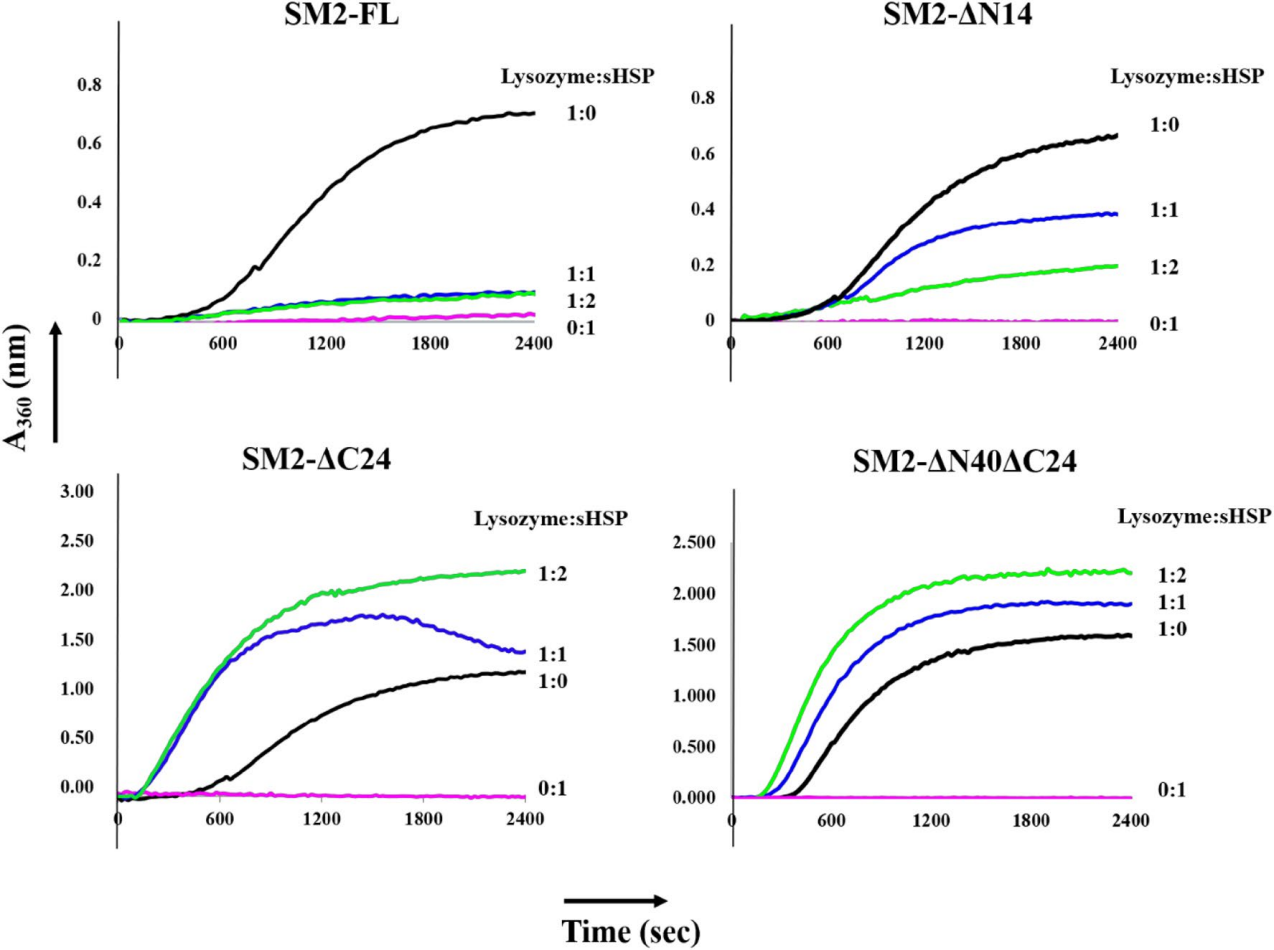
Lysozyme aggregation prevention assay: Light scattered by lysozyme observed at 360 nm at different ratios of lysozyme:SM2 constructs SM2-FL, SM2-ΔN14, SM2-ΔC24 and SM2-ΔN40ΔC24 in the presence of 90 mM DTT.

The chaperone activities of SM2 and the mutants were compared by monitoring the absorbance of light scattered by the aggregation of the model substrate lysozyme (Fig. 3) during chemically-induced denaturation using DTT. The decrease in absorbance of the scattered light in the presence of sHSP indicates less aggregation, as lysozyme is protected from chemical denaturation. The SM2 constructs were incubated with lysozyme at different molar ratios. The aggregation of SM2 constructs was not observed in the presence of dithiothreitol (DTT). Both SM2-FL and SM2-ΔN14 could protect lysozyme, however, SM2-FL offers the best protection. The SM2-ΔC24 and SM2-ΔN40ΔC24 constructs did not show any protection of lysozyme (Fig. 3). Thus, it was found that both the termini influence the chaperone activity of the protein on lysozyme. The variation in the sequence length changes the extent of protection of substrate.

### The hydrophobic surfaces vary between oligomers

SM2 has two tryptophans (Trp58 and Trp67). The fluorescence emission spectra showed an increased fluorescence intensity from the tryptophans with an increase in protein concentration (Fig. S2A). Bis-ANS is a compound that binds to the hydrophobic regions of the protein. As shown in Fig. S2B, the fluorescence intensity of bis-ANS increased with increased concentrations of SM2 constructs suggesting the hydrophobic nature of the surfaces of SM2 constructs. The fluorescence resonance energy transfer (FRET) studies carried out with SM2 constructs, incubated with various concentrations of bis-ANS (Fig. S2C), showed considerable FRET between tryptophans and bis-ANS suggesting the proximity between the two. The higher bis-ANS intensity of the higher oligomers (SM2-FL and SM2-ΔN14) reveals that more of their Trp residues are located at exposed hydrophobic regions compared to SM2-ΔC24 and SM2-ΔN40ΔC24, which form dimers in solution. This experiment showed the maximum FRET between tryptophan and bis-ANS for SM2-FL and the least for the dimers, once again revealing the role of the termini in the higher order structures and related properties of sHSPs.

The full length protein was expressed with and without the N-terminal hexa-histidine tag. Extra residues at the N-terminus are likely to influence the oligomerization and substrate specificity of sHSPs as this region is implicated in both of these properties. In the case of SM2, we observed similar behaviour for both the constructs except that the tagless construct appeared to be more heterogeneous as observed in negative stain EM images. Further structural work was carried out with the his-tagged construct. Based on this observation, the other constructs used in the present study were also expressed along with the N-terminal hexa-histidine tag and analysed.

### Crystal structure of SM2-ΔN14

**Figure 4:**
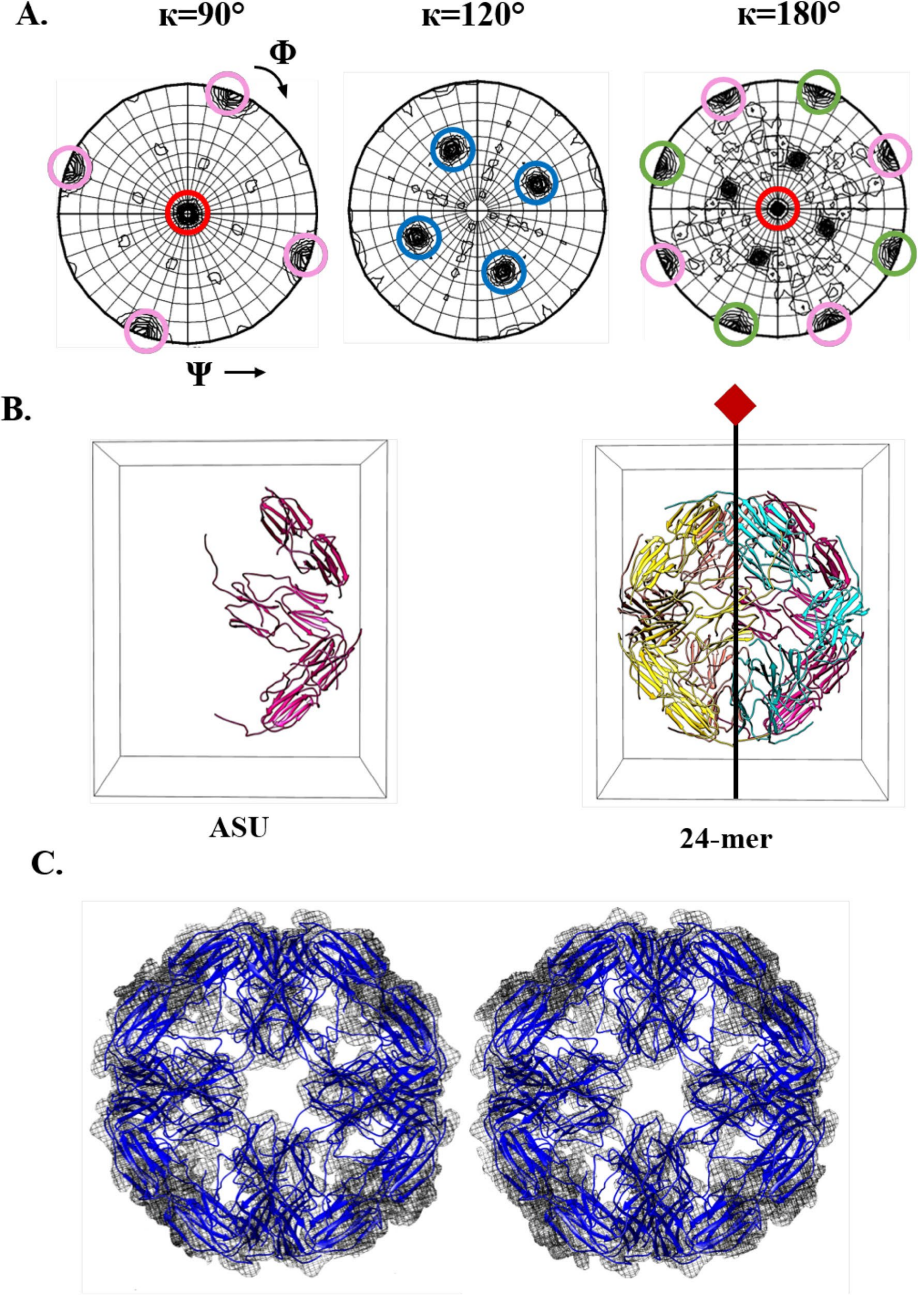
Crystallographic analysis of SM2-ΔN14 (**A**) The self-rotation function at κ = 90°, 120°, 180°. Peaks corresponding to the crystallographic fourfold axes, non-crystallographic threefold, fourfold and twofold axes are shown in blue, pink, red and green circles, respectively. (**B**) *PHASER* solution with 3 dimers in the ASU, 24-mer generated by crystallographic fourfold. (**C**) Stereo view of the electron density map (2Fo-Fc map contoured at 1.0 σ) for the 24-mer.

Of all the constructs, only SM2-ΔN14 crystallized and the crystals diffracted only to 7 Å resolution. Elongated octahedron shaped crystals of length 0.15 μm were obtained in the condition: 12 mg/ml protein concentration, 0.2 M lithium sulphate monohydrate, 0.1 M Tris pH 8.5 and 25% PEG 3350. Diffraction data were collected at the Elettra Sincrotrone to a resolution of 7 Å. The details of data collection and the data processing statistics are given in Table 2. The Matthews coefficient suggested 6 subunits in the asymmetric unit (ASU) of the I4 cell. In spite of the low resolution data, the self-rotation function exhibited clear peaks (Fig. 4A) and showed the presence of threefold non-crystallographic symmetry and additional twofold and fourfold non-crystallographic symmetries. These peaks indicated that SM2-ΔN14 has 432 symmetry.

**Table 2:** Data collection and processing statistics of SM2-ΔN14.

Parameters	SM2-ΔN14
X-ray source	Elettra, Trieste
Wavelength (Å)	0.9537
Resolution range (Å)	48.65–7.00 (7.83–7.00)
Space group	*I4*
Cell parameters: a = b, c (Å)	126.15, 152.9
Total no. of reflections	12,520 (3713)
No. of unique reflections	1915 (557)
Multiplicity	6.5 (6.7)
Completeness (%)	99.4 (100)
Mean < I > /sigma < I >	9.5 (1.3)
*R*_*merge*_ (%)	16.4 (153)
CC_1/2_	0.994 (0.628)

Values for the outer shell are given in parentheses.

The structure of SM2-ΔN14 was determined by molecular replacement (MR) using the dimer of AgsA from *S. typhymurium* (PDB code: 4ZJA)^38^ as a search model which has 24% sequence identity with SM2. Application of the crystallographic fourfold symmetry to the solution with 3 dimers resulted in a closed 24-mer with 432 symmetry (Fig. 4B), similar to the 24-mers of *S. typhymurium* AgsA (PDB code: 4ZJA^38^), *M. jannaschii* HSP16.5 (PDB code: 1SHS^9^) and *S. tokodaii* HSP14.0 (PDB code: 3VQK^39^). The solution had an LLG of 178 and TFZ of 13. Despite the data being limited to a resolution of 7 Å, the electron density map calculated after the MR solution showed clear and well-defined density for individual subunits and for the β-sheets within the subunits. Density modification, rigid body and group B-factor refinements were performed by the programs RESOLVE^40^ and *phenix.refine*^41^ implemented in PHENIX suite. The sequence of SM2 was incorporated and the model was refined to R-work and R-free values of 34.6 and 43.3%, respectively. The β-sheets were clearly visible (Fig. 4C) and the main chains of all the ACDs could be traced in the electron density. The ACD has an immunoglobulin-like fold, with two β-sheets comprising eight β-strands (β2–β9) connected by turns and loops. The electron density is absent for the first 43 residues in all the chains, indicating disorder in this region. SM2 has a long C-terminus with 31 residues, out of which only 14 residues had visible density in 5 chains and only 7 residues were visible in the 6th chain.

The current resolution does not provide atomic details of interaction of the C-terminal residues, which are known to mediate inter-dimer interactions. However, the positioning of the dimers indicates that the C-terminal regions are poised to form hydrophobic interactions with the β4–β8 cleft of the neighbouring dimers. Judging by the proximity of the clefts to the neighbouring C-termini, it is clear that a network of dimer–dimer interactions similar to those in the reported 24-mers of non-metazoan sHSPs, is formed; holding the entire 24-mer together (Fig. S3). The L-X-V patching forms the most prevalent dimer–dimer interaction in all the higher-order structures. The oligomeric state of SM2-ΔN14 in the crystal structure corroborates the SEC studies, which indicate that the protein exists as a 24-mer (Fig. 2A). The oligomer adopts an octahedral (432) symmetry. The 3 dimers in the asymmetric unit (ASU) are located along 3 of the 24 sides of the octahedron. The crystal symmetry generates a closed 24-mer.

An examination of the distribution of electrostatic potential enables us to better understand the natural formation of oligomers. The distribution of charged and hydrophobic amino acid residues provides insights into the manner in which these “natural cages” interact with non-native or damaged proteins through their hydrophobic cavities while remaining soluble themselves. In the case of SM2-ΔN14, the interior surface is hydrophobic (Fig. S4A) and the exterior surface comprises both positively- and negatively-charged amino acids (Fig. S4B) as observed in other cage-like structures of small sHSPs.

### Cryo-EM analysis of various oligomeric states of SM2-FL

Based on our SEC-MALS, DLS and negative stain EM data, SM2-FL was found to be more homogeneous than the others and was taken up for further investigations by the single particle cryo-EM analysis. Around 2,943 micrographs were collected for structural study. The details of data collection are given in Table S2. The particles showed size and structural variability with a mixed population of larger and smaller oligomers (Fig. S5A). Reference-free 2D classification of SM2-FL showed that approximately 90% of the whole dataset consisted of larger particles and 10% contained smaller particles, which are referred to as “large dataset” and “small dataset”, respectively (Fig. S5B). The particles in the major population had a diameter of 25 ± 0.75 nm and the smaller particles in the minor population had a diameter of 20 ± 0.75 nm (Fig. S5B).

**Figure 5:**
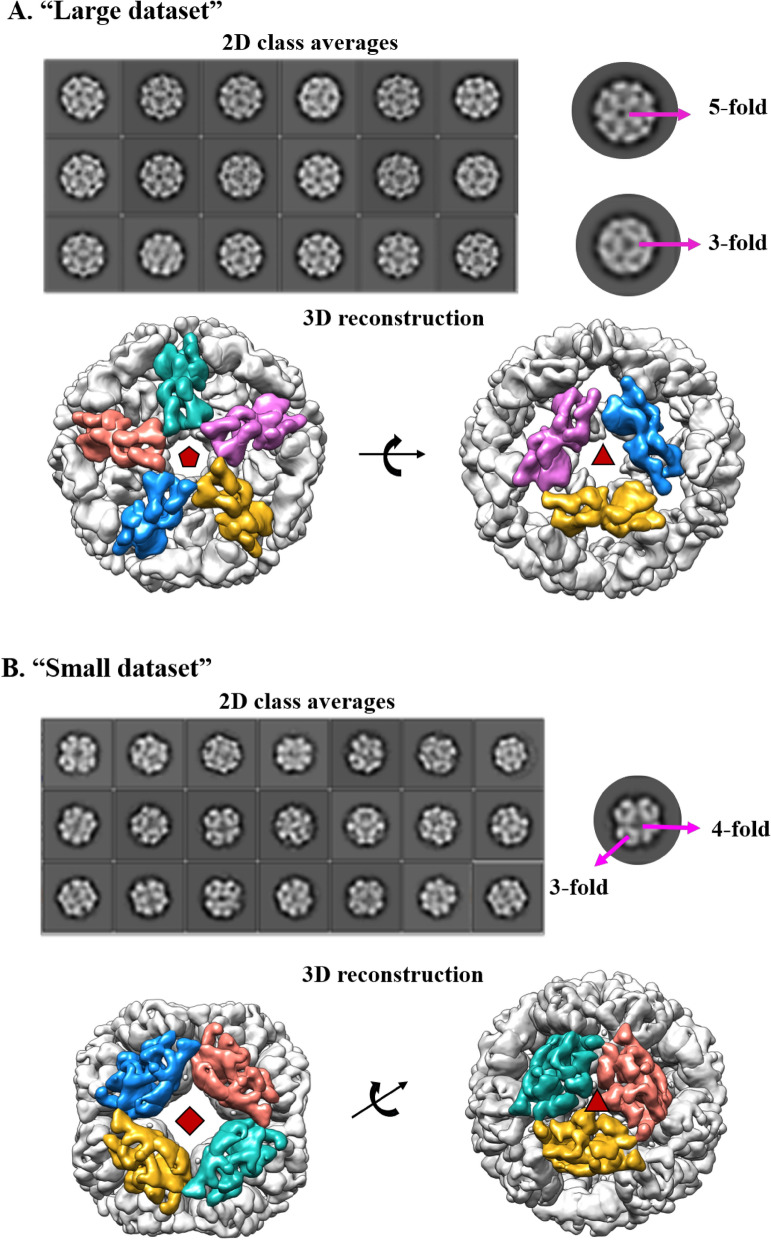
2D-classification and 3D reconstruction of (**A**) “Large dataset” highlighting the presence of fivefold and threefold symmetries. EM map viewed down the fivefold and threefold axes (**B**) “Small dataset” highlighting the presence of fourfold and threefold symmetries. EM map viewed down the fourfold and threefold axes.

The 2D class averages clearly showed the presence of fivefold, threefold and twofold symmetries without imposing any symmetry; however, the application of I symmetry (532) did not work and only D5 symmetry could be applied successfully, indicating a local threefold symmetry (Fig. 5A). The flowchart of the “large dataset” up to the refinement stage using D5 symmetry after movie processing is shown in Fig. S6A. The classes 4, 6 and 10 of the initial 3D classifications of 80,338 particles showed the best structural features. These were used for a further round of 3D classification. Finally, 57,576 particles were used for further refinement and post-processing (Fig. S6A). The 2D class averages of the “small dataset” (Fig. 5B) showed clear fourfold, threefold and twofold symmetries without imposing any symmetry. This combined 432 symmetry is observed in a number of known structures of sHSPs reported until now. The symmetry C1, i.e., onefold symmetry or no symmetry was applied to generate the initial model to know the exact symmetry of the particle. The model, once again, clearly confirmed the presence of fourfold, threefold and twofold symmetries in the particle. Octahedral (O) symmetry was further imposed to generate the initial model (Fig. S5B) and 3D classification was carried out with a pixel size of 1.2 Å. The 3D classification was carried out using 32,976 particles and the final polishing was performed with 15,258 particles (Fig. S6B).

The resolutions of the reconstructed map of the “large dataset” and “small dataset” after CTF refinement had a resolution of 8 Å and 7.7 Å, respectively, using the “gold-standard” Fourier Shell Correlation (FSC) (Fig. S7A). The EM map diameters were around 195 Å and 165 Å for the large and small datasets, respectively. A local resolution analysis indicated the resolution of the SM2-FL EM map to be between 7.5 to 8.5 Å for the “large dataset” and 7 to 8 Å for the “small dataset” (Fig. S7B). The N-termini are likely to be present inside the cage. Consistent with them being highly flexible; no density was observed for them. Rotation of the EM maps by 30° and 45° of the “large” and “small” datasets, respectively, shows the map symmetries at different angles (Fig. S7C,D).

**Figure 6:**
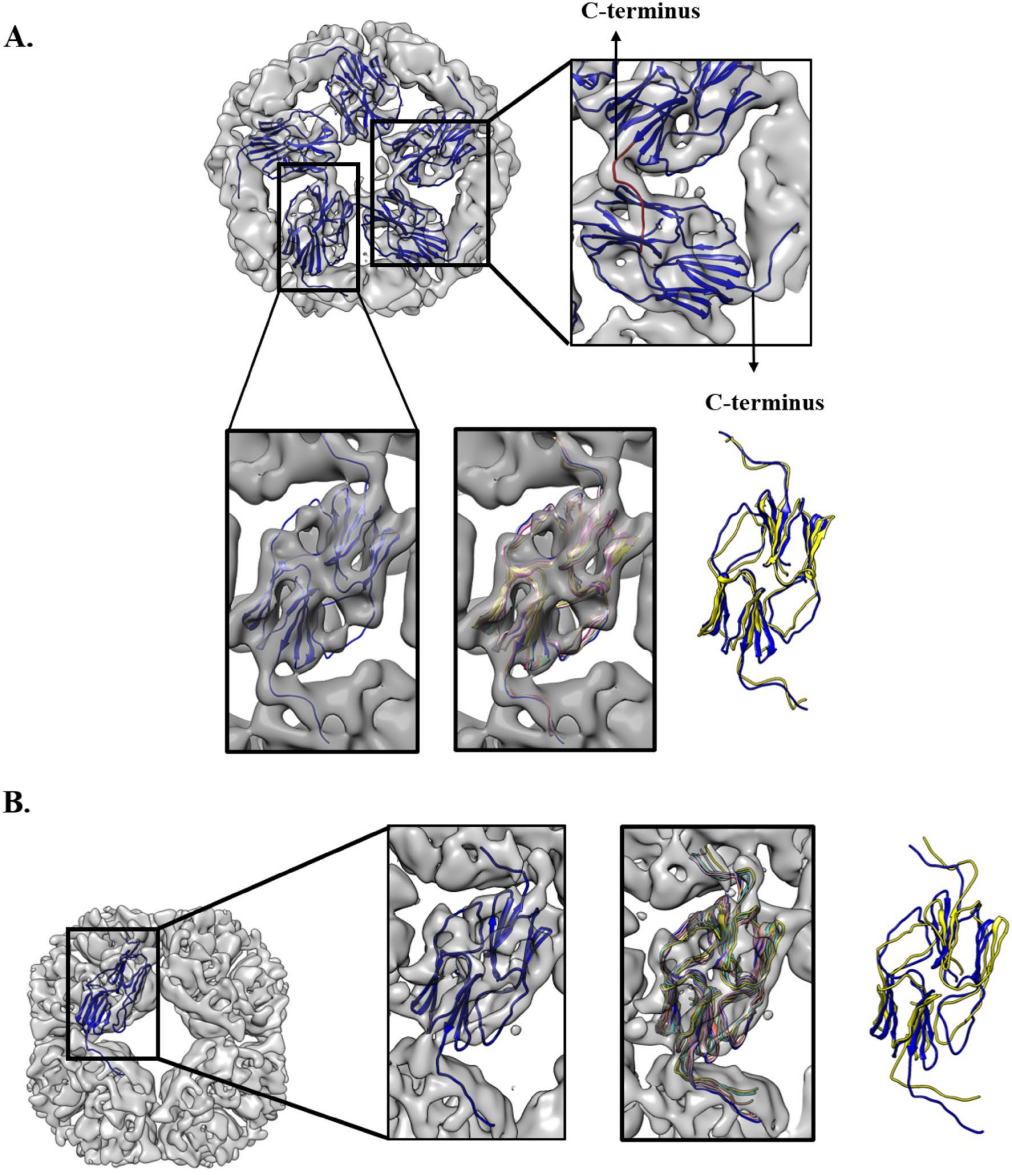
Fitting the dimers to the EM map (**A**) Docking of 5 dimers into EM map by Auto-fit using *Chimera* 1.12.0^42^ with a view down the fivefold axis of the 60-mer highlighting the interaction of the C-terminus of one subunit with a neighbouring subunit. (**B**) Docking of the crystal structure of the dimer into EM map by Auto-fit using *Chimera* 1.12.0^42^ with a view down the fourfold axis of the 48-mer. The docked dimer into the EM map with the gradual fitting of the dimerization loop into the density on successive MDFF iterations is highlighted. Initial and final fitting of the dimer are shown in blue and yellow, respectively after MDFF simulation.

The dimer of the crystal structure of SM2-ΔN14 was fitted to the 3D cryo-EM of “large dataset” (Fig. 6A) and “small dataset” (Fig. 6B) using the program *Chimera* 1.12.0^42^. For better fitting of the model to EM density, molecular dynamics flexible fitting (MDFF) simulations were carried out for the dimer and the trimer of dimers separately. As a result of simulations, the dimerization loop of the 60-mer moved by about 5 Å and shifted into the density, thus improving the fit in this region. The dimerization loop and the C-terminus are flexible and differ in the reported structures of sHSPs. This is the first instance where the EM map of a sHSP showed detailed features. In each subunit, the β-sheet regions along with a separation between them and the loops are clearly seen in the 60-mer (Fig. 6A).

The cryo-EM studies clearly showed that the “large dataset” of SM2-FL forms a cage-like particle with 532 symmetry. The particle contains 60 subunits with 30 dimers and the dimers are arranged as trimers and pentamers on the surface of the particle. Each particle of the “small dataset” contains a total of 48 subunits, or 24 dimers arranged in a cage-like assembly with 432 symmetry.

### Comparison of SM2 structures with the reported higher oligomeric structures of sHSPs

A majority of sHSPs exist as large oligomers with the dimer being the basic building block. Dynamic exchange of subunits between sHSP oligomers was reported over 20 years ago^43^. In most of the structures of sHSPs reported so far, it is observed that the density for the N-terminus is either not resolved or absent, suggesting that this region is disordered. The density of the N-terminus is visible only in the crystal structures of *Ta*HSP16.9 of *Triticum aestivum* and *Sp*HSP16.0 of *Schizosaccharomyces pombe* where the N-terminus forms α-helices and is located in the interior of sHSP oligomers. The C-termini with the I/L/V–X–I/L/V motif help in connecting the dimers in different geometries, giving rise to a variety of structures (Table S3).

The 14-residue segment of the C-terminus of SM2 has the L-X-V (Leu144-Lys145-Val146) motif, which interacts with neighbouring subunits to form large oligomers. Figure S8A shows the superposition of the dimers (residues 44–149 in each subunit) of SM2 24-mers, 48-mers and 60-mers. At the dimeric level, there is no significant difference between the ACD structures of the three oligomers. Variation is mainly seen in the C-terminal tail, which by different modes of binding with neighbouring subunits, forms various kinds of oligomers. Though the structures were determined at low resolution, it is clear that the C-terminal I-X-I motif interacted with the neighbouring molecules in a way similar to that observed in other sHSP structures.

The C-termini of all the reported sHSPs from non-metazoans lack a defined secondary structure. They are flexible and have a hydrophobic stretch that binds to a hydrophobic groove of the neighbouring subunit to form a large oligomer. A key difference between the reported oligomers lies in the hinge angle between the ACD and C-terminal extension. Variation in this angle and the direction of the C-terminus facilitate the formation of different kinds of oligomers. A few examples are given in Fig. S8B, which shows the superposition of the dimers of SM2 24-mers, 48-mers and 60-mers with other reported non-metazoan sHSPs. Variations in the orientation of the C-termini lead to assemblies of different kinds. *M. jannaschii* HSP16.5 assembles into a spherical oligomer of 24 subunits with an octahedral symmetry*.* A variant of it was reported, where on addition of 14 amino acid peptide, a stretch of human Hsp27 was inserted into the N-terminal region of *M. jannaschi*i *j*HSP16.5. This insertion changed the oligomer from 24-mers to 48-mers. Variation was seen only in the directions of the C-terminal regions of the two oligomers (Fig. S8C), leading to changes in the oligomeric size. Similar changes were observed in SM2 (Fig. S8A).

The eukaryotic sHSPs, *T. aestivum* HSP16.9 and *S. pombe* HSP16.0, form oligomers but not as closed cage-like structures. *T. aestivum* HSP16.9 consists of two stacked hexameric rings whereas *S. pombe* HSP16.0 forms an elongated 16-meric oligomer. On comparing the C-terminal regions of both the structures with those of the 24-mers of SM2, it is clear that the different oligomeric architectures arise from different orientations of the C-terminal tail relative to the ACD (Fig. S8B). The C-termini of *S. pombe* HSP16.0 dimers are in the same direction whereas those of *T. aestivum* HSP16.9 and SM2 dimer are in opposite directions (Fig. S8B).

### Comparison of the conserved hexameric assembly (trimer of dimers) in sHSP oligomers

Upon analysing the available sHSP oligomeric structures, it was observed that hexamers (trimer of dimers) with 32 symmetry are present in a majority of them. Conservation of this hexameric assembly has been observed in all the three oligomers of SM2 despite the variations in oligomeric composition and sizes. Figure S9A shows the superposition of the hexamers of SM2 24-mers, 48-mers and 60-mers. On superposition of one of the dimers of the trimer, shifts in the other dimers of the trimer were observed. This variation was because of the interaction of the C-termini with the ACD of neighbouring trimers, tetramers and pentamers in the 24-mers, 48-mers and 60-mers of SM2, respectively.

The hexamers of the 24- and 48-mers of SM2 and of *M. jannaschii *HSP16.5 were compared (Fig. S9A,B). The C-terminus in one of the subunits of each dimer interacts with the other dimers within the hexamer, stabilizing the hexameric sub-assemblies while the C-terminus of the other subunit of the dimer is involved in interactions between the hexamers. Different extents of bending of the hexamers and the C-termini were observed for the formation of the 48-mers and 24-mers. The flexibility and directionality of binding of the C-termini play a crucial role in forming various oligomers. The presence of hexamers is also observed in two other higher oligomeric structures of sHSPs: *Ta*HSP16.9 from *T. aestivum* and M3 from *Mycobacterium marinum,* which form different types of 12-mers. *T. aestivum* HSP16.9 forms a double disc-like structure with three dimers in each disc. The N- and C-terminal interactions across the dimers stabilize the two discs, whereas in M3, both the dimerization loop and the C-terminus mediate dimer–dimer interactions. When one dimer of the hexamer of these two structures is superposed on one of the dimers of the hexamer of SM2 24-mers, a shift in the other two dimers is clearly visible (Fig. S9C). The side view clearly shows different extents of bending of the trimers and the C-termini for the formation of different oligomers. The curvature of the hexamer in the enclosed dodecameric M3 is more than that of the *T. aestivum* HSP16.9 hexamer. Hexamers of both the 12-mers bend more than the 24-mers structure of SM2. The SM2 structure has hexameric sub-assemblies very similar to those of other reported sHSP structures. The variation is mainly seen in the C-terminus and the curvature of the hexamers in different oligomers.

### Comparison of the whole assemblies of 24-mers, 48-mers and 60-mers of SM2

**Figure 7:**
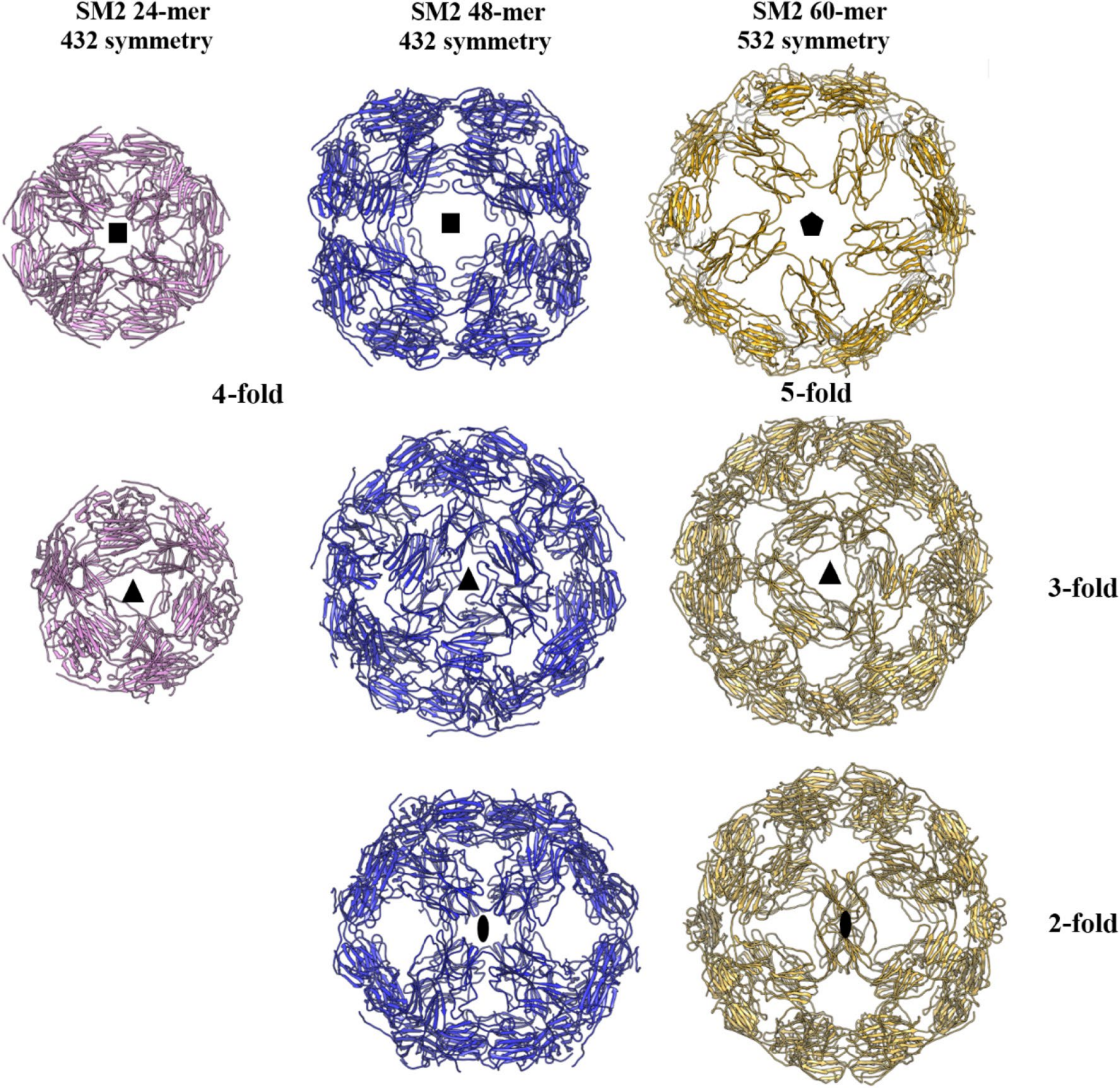
SM2 24-mer, 48-mer and 60-mer viewed down their respective symmetry axes.

The SM2 constructs form 3 different oligomers with different symmetries, sizes and shapes with the same dimeric building block (Fig. 7). The 24-mers and 48-mers have the same symmetry of 432, but the 60-mers formed by 532 symmetry. In the 24-mers, the twofold relates two subunits of the dimer and in the 48-mers, the twofold relates two dimers. In all the three structures, the hexameric assembly is observed. The fivefold symmetry in the 60-mer structure was observed for the first time in sHSPs.

### Dynamic oligomerization and activity of SM2

In the present study, we could capture a few higher oligomeric forms of SM2. In an earlier report^35^, it was observed that large oligomers of SM2 (~ 32-mers and dodecamers) dissociate into smaller oligomers (dimers or trimers) at higher temperatures (45 °C); and in the presence of heat-denatured substrates, malate dehydrogenase and luciferase, stable and soluble complexes of SM2 + substrate bigger than the SM2 oligomers were seen. The authors propose that under heat stress, large oligomers of SM2 dissociate into smaller oligomers with exposed N-termini, bind to the client proteins and reassociate to form large oligomers again, as proposed for many other sHSPs. Since we observed large oligomers, 48-mers and 60-mers, with long channels through the symmetry axes, there is also the possibility that some of the denatured substrates pass through these channels and bind to the N-termini of SM2 located at the core of the cage-like structures, as in the case of HSP16.5 of *M. jannaschii* in complex with destabilized T4 lysozyme^22^. Cryo-EM analysis revealed that T4 lysozyme is located inside the core of 24- and 48-meric structures of HSP16.5, interacting with the N-termini. The authors mentioned the possibility of the denatured substrates passing through the channels around the symmetry axes to the core of the cage. Similar situation is likely to happen in SM2 for which we observed different oligomeric structures with cavities ranging from 25 to 30 Å in diameter around the 3-folds and fivefold axes and about 40 Å around the fourfold axes. We did not observe any smaller oligomers as we did not perform activity studies at higher temperatures, i.e., around 45 °C. The lysozyme aggregation prevention assay for SM2 was carried out at 37 °C which clearly showed that SM2 was active, as it could suppress the aggregation of chemically denatured lysozyme. In this case, not the heat, but the presence of the denatured substrate could have activated SM2. Both the mechanisms are possible, that the oligomer dissociated into smaller oligomers as in the case of the heat denatured substrates^35^, or the substrate interacted directly with the larger oligomers of SM2.

## Conclusions

We present the structural features of SM2 constructs and compare them with other available structures of this class of proteins. SM2-ΔN14 crystallized as a 24-mer with 432 symmetry, similar to the 24-mers of *S. typhymurium* AgsA*, M. jannaschii *HSP16.5 and *S. tokodoii* HSP14.0. The structures of two different oligomers, a 48-mer and a 60-mer, of SM2 were determined by cryo-EM. These particles with different sizes/diameters were separated from the micrographs of the same sample and processed. The symmetries of the particles: 432 for the 48-mers and 532 for the 60-mers were clearly visible in the 2D and 3D class averages even before any symmetry was applied. The structures were determined at 8 Å by fitting the dimers obtained from a low-resolution crystal structure of SM2. The 48-mer is similar to a variant of *M. jannaschii* HSP16.5^44^, which also has 432 symmetry. As already established, the dimer is the building block that assembles into a variety of observed oligomers of sHSPs made of 12-, 16-, 18-, 24-, 32- and 48-mers. Here, we report for the first time, the formation of a 60-mer in sHSPs as an assembly of 30 dimers. These are the first structures of any viral sHSP to be reported and the 60-mer is the largest oligomer of the reported sHSPs. The C-terminus plays an important role in the formation of various oligomers. The hexameric assembly observed in the oligomers of different symmetries of sHSPs was found to be similar. The flexibility of the C-terminal region combined with slight differences in the arrangement of dimers in the hexameric assembly has enabled the variation in oligomerization. The cryo-EM maps of SM2 obtained in the present study clearly showed the details of the secondary structures at the subunit level. The reported cryo-EM structures of sHSPs from *M. tuberculosis*^45^ and *A. thaliana*^46^ are at 15 and 10 Å respectively, and the model that was fitted to the density was that of *Triticum aestivum* sHSP. Structural features within the subunits were not clearly visible.

The following functional insights provided by the present work on SM2 support the previous observations on sHSPs. The N-terminus is crucial for sHSP activity as deletion of 14 N-terminal residues reduced the interaction of SM2 with the model substrate, lysozyme. The C-terminus interacts with the neighbouring dimers by changing its direction and generates various oligomeric states enabling them to recognize different substrates. This flexibility of the C-terminus is thus responsible for the unique property of sHSPs to have a wide variety of substrates. In the case of SM2, we observed that the C-terminal deletion mutant forms only dimers and has no chaperonic activity indicating that the inability of sHSPs to form higher oligomers abolishes their activity. In a previous study, complexes of SM2 with model substates MDH and luciferase were shown to exist as higher oligomers. The presence of a wide range of SM2 structures of various sizes and symmetries that we observed in solution either by SEC or DLS or negative stain EM suggests that SM2 can interact with a variety of substrate proteins, either its own or from the host. The in vivo role of viral sHSPs is yet to be explored.

Our studies confirmed the highly polydisperse and heterogenic nature of sHSPs. Nevertheless, we could study the structures of three types of particles of SM2, which revealed new features related to the oligomerization and symmetry of sHSPs. One of the forms of SM2 has icosahedral symmetry, the most prevalent symmetry of the viral capsids. Further investigations are required to ascertain whether this has any relevance to the viral origin of the sHSPs.

## Methods

### Cloning

The gene corresponding to SM2 (GenBank ID: GU071096.1) was codon optimized and synthesized by Genscript, USA. The gene for full length SM2 (SM2-FL) was cloned in pUC57 vector between the *NheI* and *XhoI* restriction sites. Pfu DNA polymerase was used to amplify all the constructs. Following polymerase chain reaction (PCR) amplification of the gene, both the gene and vector were digested by the restriction enzymes (New England Biolabs, USA) *NheI* and *XhoI*. The digested genes and vectors were ligated by T4 DNA ligase. The sequences of all the clones subsequently obtained were confirmed by DNA sequencing. The constructs were ligated to pET-28a vector, which has an N-terminal hexa-histidine tag that incorporates 23 residues from the vector, including an N-terminal hexa-histidine tag.

The following constructs were cloned:

1. Full length SM2 Tagged (SM2-FL)
2. Deletion of 14 amino acids from N-terminus (SM2-ΔN14)
3. Deletion of 24 amino acids from C-terminus (SM2-ΔC24)
4. Deletion of 40 amino acids from N-terminus and 24 amino acids from C-terminus (SM2-ΔN40ΔC24).

### Expression and purification of SM2 constructs

The clones were transformed into *E. coli* BL21 (DE3) expression cells and grown on solid Luria–Bertani media containing 30 µg/ml of the antibiotic kanamycin. A single colony of the transformed BL21 (DE3) cells was used to inoculate 10 ml of primary LB media, which was subsequently grown overnight. Eight hundred ml of secondary media was inoculated with 1% of the overnight primary culture and grown at 37 °C until the optical density (OD) at 600 nm reached 0.6. The expression of sHSPs was induced by the addition of 0.3 to 0.5 mM Isopropyl β-D-1-thiogalactopyranoside (IPTG) for various constructs and the culture was further grown at 37 °C for 4 h. Then, the culture was centrifuged at 5000 rpm for 15 min to separate the cells from the media and pelleted. The pellet was resuspended in lysis buffer containing 20 mM Tris and 200 mM NaCl at pH 8.0. The cells were lysed by sonication and centrifuged to separate the cell debris from the soluble fraction. The supernatant was loaded onto Nickel-NTA resin, which had been pre-equilibrated with lysis buffer. The proteins were eluted from the column by imidazole gradient from 30 to 500 mM. The elution fractions containing the protein were identified by sodium dodecyl sulfate–polyacrylamide gel electrophoresis (SDS-PAGE). This technique was also used to confirm the presence of the protein and its purity. The molecular masses were confirmed by matrix-assisted laser desorption/ionization-time of flight (MALDI-TOF) mass spectrometry. Protein concentration was determined by measuring the UV absorbance at 280 nm.

The protein was kept in the buffer containing 20 mM Tris and 200 mM NaCl at pH 8.0 for further experiments.

### Size exclusion chromatography-multi angle light scattering (SEC-MALS)

SEC–MALS experiments were performed using the Superose-6 10/300 GL, GE column, at room temperature (25 °C). Bovine serum albumin (BSA) was used as a calibration standard with the Wyatt 762-TS miniDAWN TREOS instrument for all full length and deletion constructs. 1 mg/ml (100 μl) of all the constructs were centrifuged at 14,000 rpm for 10 min before injecting them into the column. The flow rate was maintained at 0.2 ml/min for each sample.

### Dynamic light scattering (DLS)

To determine the hydrodynamic radii and polydispersity of different constructs, DLS experiments were performed with Xtal SpectroSize 300 at 25 °C with 0.5 mg/ml of the samples. The hydrodynamic radii and polydispersity index data are shown in Table S1.

### CD spectroscopy

The CD spectra of sHSPs were recorded on a Jasco J-715C spectropolarimeter at a scan rate of 50 nm/min, response time of 4 s and a bandwidth of 2 nm. The experiments were carried out in quartz cuvette with 1 cm path length at 25 °C.

### Intrinsic tryptophan fluorescence

The intrinsic tryptophan fluorescence spectra of 3 μM protein were recorded using a Jasco FP-6300 spectrofluorometer. The excitation wavelength was set to 280 nm and the emission spectra were recorded from 300 to 550 nm. The total reaction volume was 200 μl and the reaction mixtures were incubated at 25 °C for 30 min before recording the spectra. The run was repeated three times.

### Interaction with bis-ANS and fluorescence resonance energy transfer (FRET)

The surface hydrophobicity of the protein was probed with bis-ANS, which is a compound that binds to the hydrophobic regions of proteins. 3 μM of bis-ANS was incubated with different concentrations of SM2 constructs and fluorescence spectra were recorded between 400 to 600 nm using an excitation wavelength of 395 nm. Both the excitation and emission band passes were 2.5 nm. A scan speed of 100 nm/sec was maintained.To probe the interaction between bis-ANS and the protein, FRET was performed using 3 μM of protein incubated with 1 μM, 2 μM and 5 μM bis-ANS at 25 °C and spectra were recorded between 310 and 550 nm using an excitation wavelength of 295 nm. A scan speed of 100 nm/s was maintained. The runs were repeated three times.

### Chaperone activity assay

Hen egg-white lysozyme, a 14.3 kDa enzyme, is one of the readily available proteins for aggregation study. 15 µM of lysozyme in 50 mM sodium phosphate buffer, pH 7.4, was denatured with 90 mM DTT at 37 °C. The aggregation of lysozyme was monitored by a spectrophotometer, by measuring the absorbance of the scattered light at 360 nm. An increase in aggregation over time shows an increase in absorbance. The aggregation of lysozyme in the presence of sHSP was measured for 30 min at 37 °C. The procedure was repeated for various molar ratios of sHSP: lysozyme.

### Crystallization, data collection, structure solution and refinement

Crystallization trials for all the constructs were set up by the hanging drop vapour diffusion method with TTP labtech mosquito LCP machine. Initial screening was performed using the Hampton Research, Jena Bioscience and MIDAS crystallization kits. Different concentrations of protein ranging from 5 to 20 mg/ml were used for setting up the trials.

Diffraction data were collected on the XRD2 beamline of the Elettra Sincrotrone at Trieste, Italy to 7 Å resolution. 180 frames were collected at a wavelength of 0.9537 Å with an oscillation angle of 1° per image using a Dectris Pilatus3-6 M detector. The diffraction images were processed by *XDS*^47^ and the scaling of integrated intensities was carried out using the program *AIMLESS*, which suggested the space group to be I4. The data collection and processing statistics are given in Table 2. The *CCP4i2* suite was used for further computations: self-rotation and MR were carried out using MOLREP and *Phaser*. Refinement was carried out by PHENIX suite.

### Negative staining

Carbon-coated TEM grids EMS (Electron Microscopy Sciences, USA) were first negatively glow-discharged at 20 mA for 25 s in a GloQube glow discharge system (Quoram technologies) to create a hydrophilic surface for the even distribution of protein particles. 3.5 μl of the protein sample (0.1–0.5 mg/ml) were loaded on the grid and allowed to stay for 1 min for absorption by carbon. Digital micrographs were recorded at 120 kV, at calibrated 75 k magnification with the defocus range between -1 and -2 µm under low-dose mode on a FEI Tecnai 12 BioTwin transmission electron microscope fitted with a LaB6 (lanthanum hexaboride crystal) filament. An Olympus VELITA (2 K × 2 K) CCD camera was used for data collection. The beam was aligned and the eucentric height and astigmatism were adjusted before imaging. Well-separated particle images on micrographs were manually selected using Boxer from *EMAN2.1*^48^ software package.

### Cryo-EM sample preparation and imaging

Purified protein samples at a concentration of 1–3 mg/ml were used for cryo-EM data collection. The Quantifoil R2/1 300 mesh holey carbon grids were first glow discharged for 90–120 s at 20 mA current using a GloQube glow discharge system (Quoram Technologies) for uniform distribution of particles on the grid. Three microlitres of the protein sample (3 mg/ml) were loaded on the grid and the grid was automatically blotted for 3 s, followed by plunge-freezing in liquid ethane using a FEI Vitrobot Mark IV plunger.

Vitrified grids were used for cryo-EM data acquisition and data collection was performed using the Thermo Scientific™ Talos Arctica TEM at 200 kV equipped with a K2 Summit direct electron detector (Table S2). The images were collected automatically using the *LatitudeS* automatic data collection software (Gatan, Inc.) at a nominal magnification of 42,200 × corresponding to a pixel size of 1.2 Å at the specimen level. A total of about 40 e^−^/Å^2^ electron doses were used and fractionated to 2 e^−^/Å^2^ over 20 frames; the images had a defocus range from − 1.25 to − 3.5 μM. Statistics are shown in Table S2. A total of 2,943 micrographs were collected for SM2-FL for cryo-EM studies.

### Image processing

Data processing was performed using the *RELION 3.0*^49^ and *EMAN2.1*^48^ software packages. The *MotionCor2* program^50^ was used for correcting the beam-induced sample motion recorded on dose-fractionated movie stacks. The motion-corrected micrographs were manually evaluated and the best micrographs were considered for further processing by *RELION 3.0*^49^. The defocus and beam astigmatism were determined using the CTFFIND4.1 program^51^.

From the whole dataset, 1,597,888 particles were extracted with a box size of 280 pixels, down-scaled three times, subjected to 2D classification and sorted into 100 classes. The particle diameter used was 260 Å. After a few rounds of 2D reference-free classification, 256,473 particle projections from well-defined averages were selected. At this point, as it was observed that the sample consisted of a major population of larger particles and a minor population of smaller particles, the total population was divided into a “large dataset” and a “small dataset” depending on the particle size and symmetry. The “large dataset” had 80,338 particles and was further processed for 3D-classification. Out of this, 24,789 particles were used for generating the initial model. After 3 rounds of 3D classification using the initial model, 57,576 particles were used for further 3D refinement. The “small dataset” was re-extracted with a box size of 264 pixels and further subjected to 2D-classification with 32,976 particles, with a final particle diameter of 250 Å (Fig. S5B). A 3D initial model was generated using 6,800 particles and after 2 rounds of 3D classification, 15,247 particles were used for further 3D refinement. The final refined density was further filtered at the correct resolution with a map that masks the solvent in the post-processing procedure of *RELION*^52^. Additionally, the density was sharpened according to an automatically-estimated B-factor^53^. The parameters used for 3D refinement were not changed during the resolution test. The resolution of the 3D model was determined as the point where the FSC curve falls below the threshold of 0.143^53^.

### Local resolution estimation

The program *ResMap*^54^ is used for calculating local resolution using *RELION 3.0*^49^ software. The input is Refine-3D half map to ‘Local resolution’ option along with the mask used. It calculates and reports the local resolution at each voxel of the input maps^55^. At any voxel, the local resolution is the wavelength of the highest local spatial frequency that is statistically significant above noise. The *ResMap* software calculates local resolutions within a range at a given step size, both specified by the user^55^.

### Fitting of the crystal structure to the EM map

The dimer (residues 44–149 in each subunit), of the crystal structure of the 24-meric SM2-ΔN14, was used for rigid body fitting to the 3D cryo-EM map using the UCSF *Chimera* program^42^. The dimer was initially fitted to the cryo-EM map manually. In this manual fitting, the ACD fitted well in the density but the dimerization loop deviated from the density. A rigid body fitting using “fit in map” of the *Chimera* 1.12.0 software improved the fit of the dimer (Fig. 6A,B). Automatic and interactive fitting of the structure to the EM density using the ‘Fit in map’ command of the 3D viewing *Chimera* 1.12.0 software was performed. Individual dimers were fitted to the map to get the higher oligomeric structures. The cross-section of 3D volume and core diameter were calculated using UCSF *Chimera* 1.12.0^42^.

The MDFF method was used to fit atomic structures into low resolution EM as described in Trabuco et al.^56^. We generated all MD trajectories using *NAMDv2.8*^57^ and the *CHARMM* force field^58^. *VMDv1.9* was used for system creation and protein rendering^59^.

## Supplementary Information

Supplementary Information.
