# Supplementary material for: Multiple nanocages of a cyanophage small heat shock protein with icosahedral and octahedral symmetries
Source: Sci Rep. 2021 Oct 25;11:21023. doi: 10.1038/s41598-021-00172-2 (PMC8546028; doi:10.1038/s41598-021-00172-2)
Supplement: Supplementary file 1 — Supplementary Information. [file 41598_2021_172_MOESM1_ESM.docx]

**Multiple nanocages of a cyanophage small Heat Shock Protein**

**with icosahedral and octahedral symmetries**

Sreeparna Biswas, Priyanka Garg, Somnath Dutta* and Kaza Suguna*

Molecular Biophysics Unit, Indian Institute of Science, Bangalore – 560 012, India

*Corresponding authors (E-mail: somnath@iisc.ac.in, suguna@iisc.ac.in)

**Supplementary**

**Table S1:** Hydrodynamic radius (Rh) and % polydispersity of different SM2 constructs from DLS experiment.

| **Construct** | **Dynamic light scattering (DLS)** | |
| --- | --- | --- |
|  | **Rh (nm)** | **%Polydispersity** |
| SM2-FL | 12.0 (0.6) | 17.7 (2.8) |
| SM2-ΔN14 | 10.3 (0.8) | 57.0 (4.5) |
| SM2-ΔC24  (Dimer) | 8.1 (0.4) | 14.0 (3.8) |
| SM2-ΔN40ΔC24  (Dimer) | 7.7 (0.1) | 13.2 (2.2) |

Average Rh values and % of Polydispersity 25 ℃. The standard errors are given in parentheses.

**Table S2:** Cryo-EM data collection parameters.

| **Data Collection** | |
| --- | --- |
| Microscope Type | Talos Arctica 200 kV Cryo-EM (IISc, Bangalore) |
| Voltage (kV) | 200 |
| Dose (e-/ Å2) | 40 |
| Detector | K2 Direct electron detector counting mode |
| Pixel size (Å) | 1.2 |
| Defocus range (μm) | -3.5 to -1.25 |
| Electron Gun | Field emission gun |
| C2 aperture | 70 |
| Electron dose per frame (e^-^/ Å^2^) | 2 |
| Data Processing | RELION-3.0, EMAN2.1 |
| Magnification | 42000X |

**Table S3:** Structural data of sHSPs.

**X-ray crystallography data**

| **Protein** | **Origin** | **PDB** | **Residues traced in a subunit** | **Resolution**  **(Å)** | **Subunits in the oligomer (in solution)** |
| --- | --- | --- | --- | --- | --- |
| Hsp16.5 | *M. jannaschii* | 1SHS | 33–147 | 2.9 | 24 (24) |
|  |  | 4ELD | 45-161 | 2.7 | 48 (48) |
| Hsp16.9 | *Wheat (T. aestivium)* | 1GME | 2 (43^a^)–151 | 2.7 | 12 (12) |
| Hsp16.0 | *S. pombe* | 3W1Z | 10-143 | 2.4 | 16(16) |
| Hsp14.0 | *S. tokodaii* | 3VQK | 17-123 | 4.5 | 24(24) |
| M3 | *M. marinum* | 5ZS3 | 22-116 | 2.0 | 12(12) |
| Sip-1 | *C. elegans* | 4YDZ | 18-152 | 3.6 | 32(24-32) |
| AgsA | *S. typhimurium* | 4ZJA | 40-145 | 4.1 | 24(24) |
|  |  | 4ZJD | 40-132 | 7.5 | 18(12) |
| SM2 | *Synechococcus* phage S-ShM2 (SM2-ΔN14) | 7CK4 | 45-150 | 7.0 | 24(24) |

**EM data**

| **Protein** | **Origin** | **PDB** | **EMDB accession number** | **Resolution (Å)** | **Subunits in the oligomer (in solution)** |
| --- | --- | --- | --- | --- | --- |
| ACR1 (Hsp16.3) | *M. tuberculosis* | 2BYU* | 1149 | 16.5 | 12 (12) |
| Hsp26 | *S. cerevisiae* | 2H50* | 1221 | 10.8 | 24 (24–32) |
|  |  | 2H53* | 1126 | 11.5 | 24 (24–32) |
| HspB5 (αB-crystallin) | Human | Not available | 1776 | 20.0 | 24 (24–32) |
| Hsp21 | *A. thaliana* | 5NMS* | 3459 | 10.0 | 12 (12) |
| SM2 | *Synechococcus* phage S-ShM2 (SM2-FL) |  |  | 7.72 | 48(>40-mer) |
|  |  |  |  | 8.0 | 60(>40-mer) |

*: Coordinates in these files are of the wheat *Ta*Hsp16.9 ACD domain fitted into the EM density.

^a^: Six subunits of the dodecamer include the complete polypeptide (residues 2–151), while the N-terminal 42 residues are missing in the other six subunits.

**
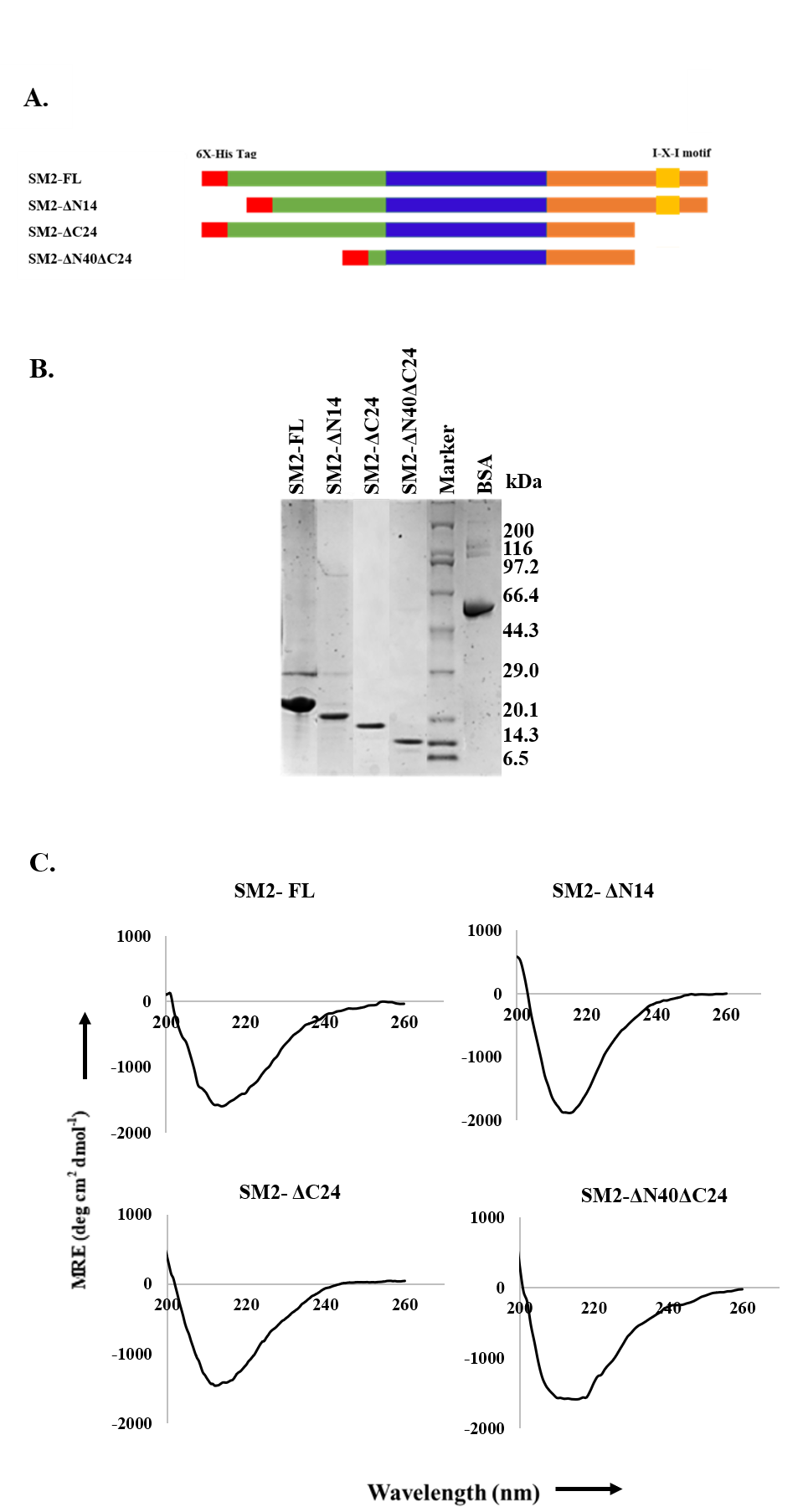
**

**Fig S1.** SM2 constructs with SDS-PAGE and CD profiles (**A**) Schematic drawing of SM2 constructs. The hexa-histidine tag is highlighted in red and the I-X-I motif in yellow. The N-terminal segment is in green, ACD in blue and the C-terminus in orange. (**B**) 12% SDS-PAGE gel. The ImageJ software^1^ was used to calculate the molecular weight and determine the purity of the samples. The molecular weights for SM2-FL, SM2-ΔN14, SM2-ΔC24 and SM2-ΔN40ΔC24 constructs were calculated to be 20.9 kDa, 18.71 kDa, 17.65 kDa and 13.97 kDa, respectively, based on the positions of the constructs with respect to the marker in the gel. The calculated values are close to the theoretical value of the masses given in Table 1. The purity of individual protein samples was further checked by finding the band intensities in comparison with the intensity of the whole lane in which they are loaded. The SM2-FL, SM2-ΔN14, SM2-ΔC24 and SM2-ΔN40ΔC24 constructs were found to be 89%, 93%, 99% and 98% pure, respectively. (**C**) CD profiles of SM2 constructs.


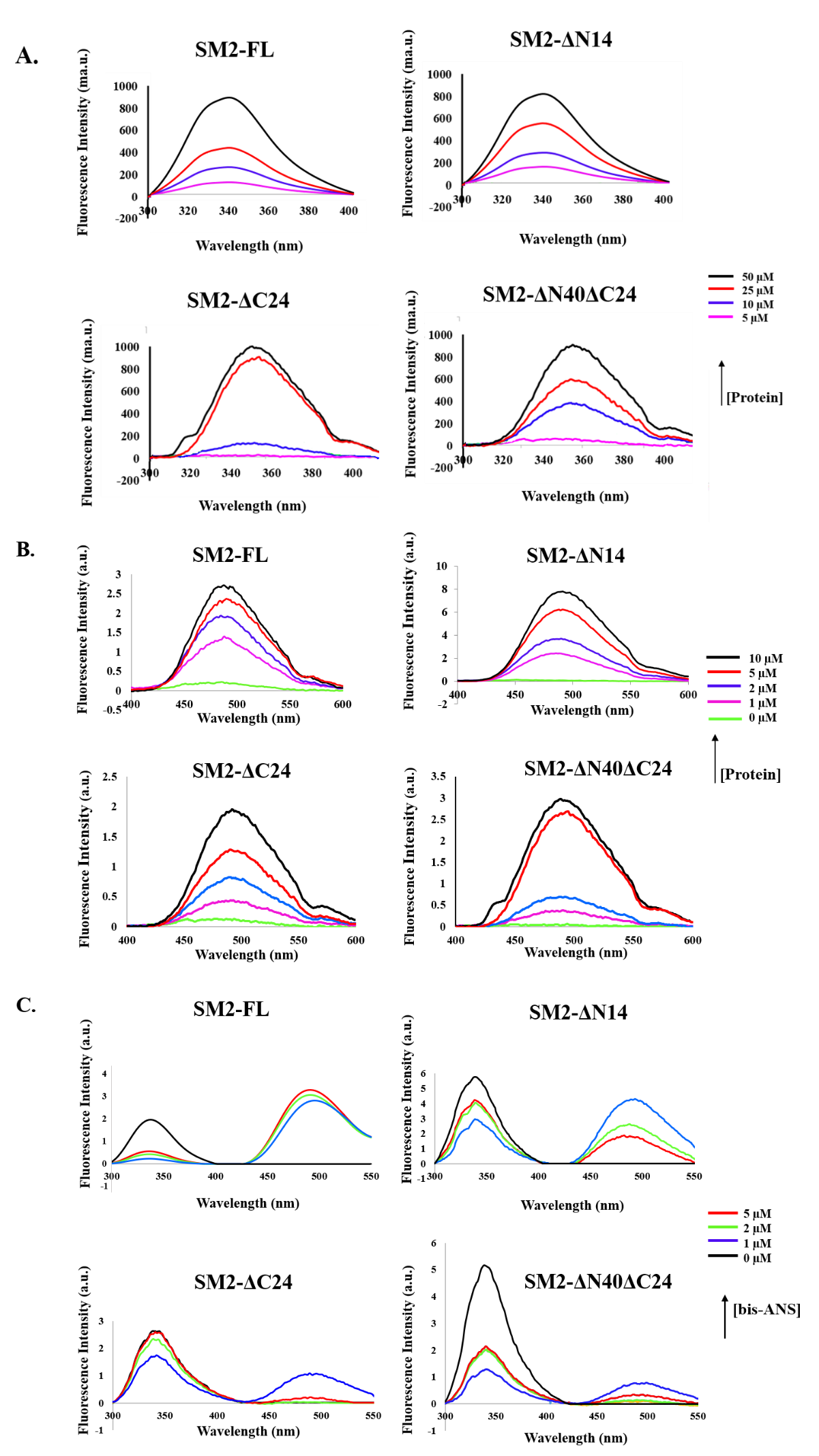


**Fig S2:** Fluorescence studies of SM2 constructs (**A**) Intrinsic tryptophan fluorescence spectra and (**B**) Fluorescence intensity of bis-ANS at different protein concentrations and (**C**) FRET study between tryptophan and bis-ANS of SM2 constructs.

**
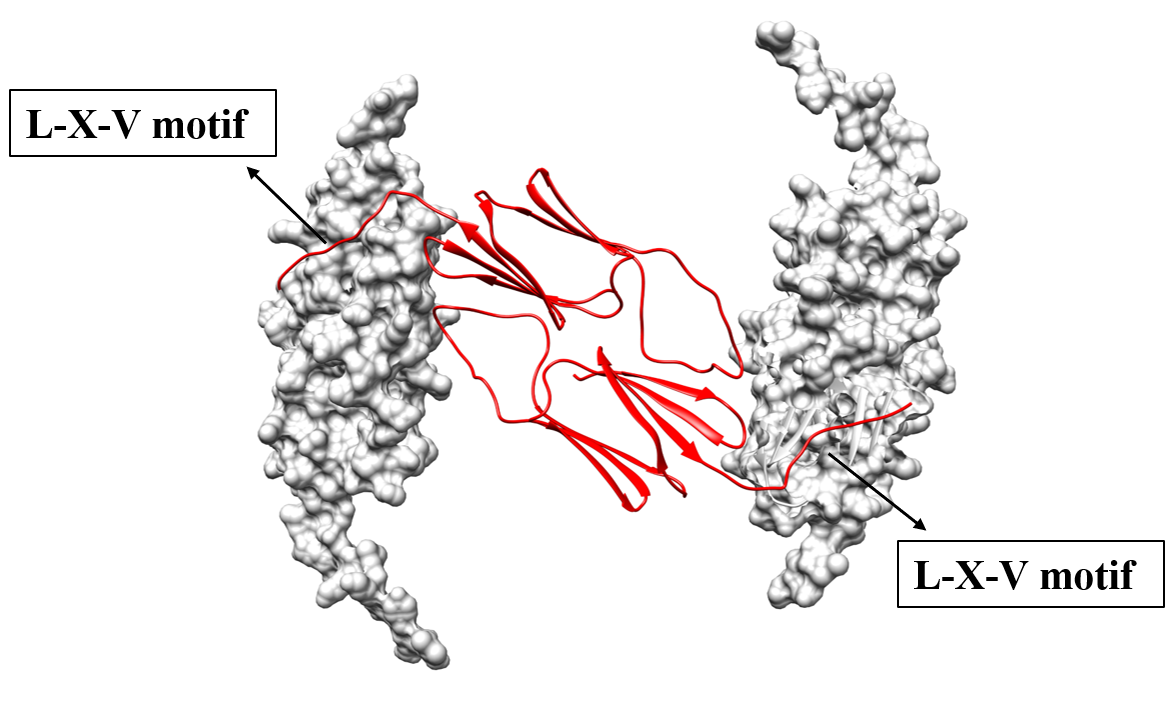
**

**Fig S3.** Interaction of L-X-V motif with the neighbouring dimer.


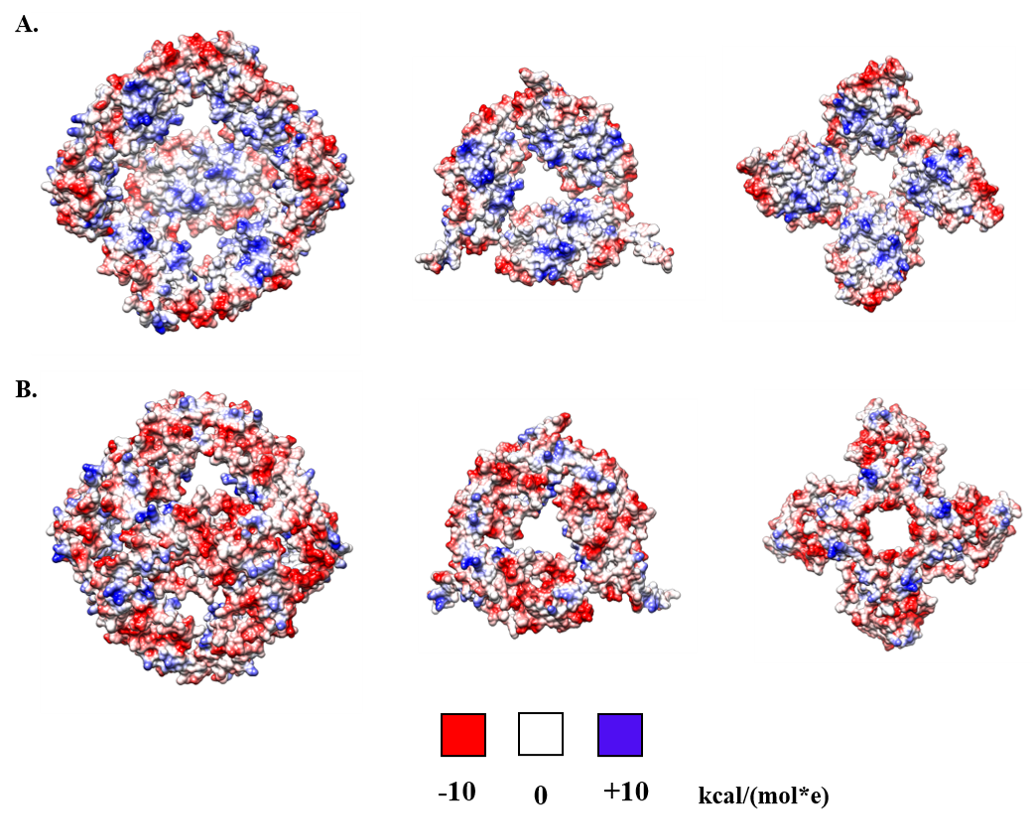


**Fig S4.** Electrostatic surface representation of different faces of the 24-mer of SM2-ΔN14 (**A**) Interior surface and (**B**) Exterior surface.


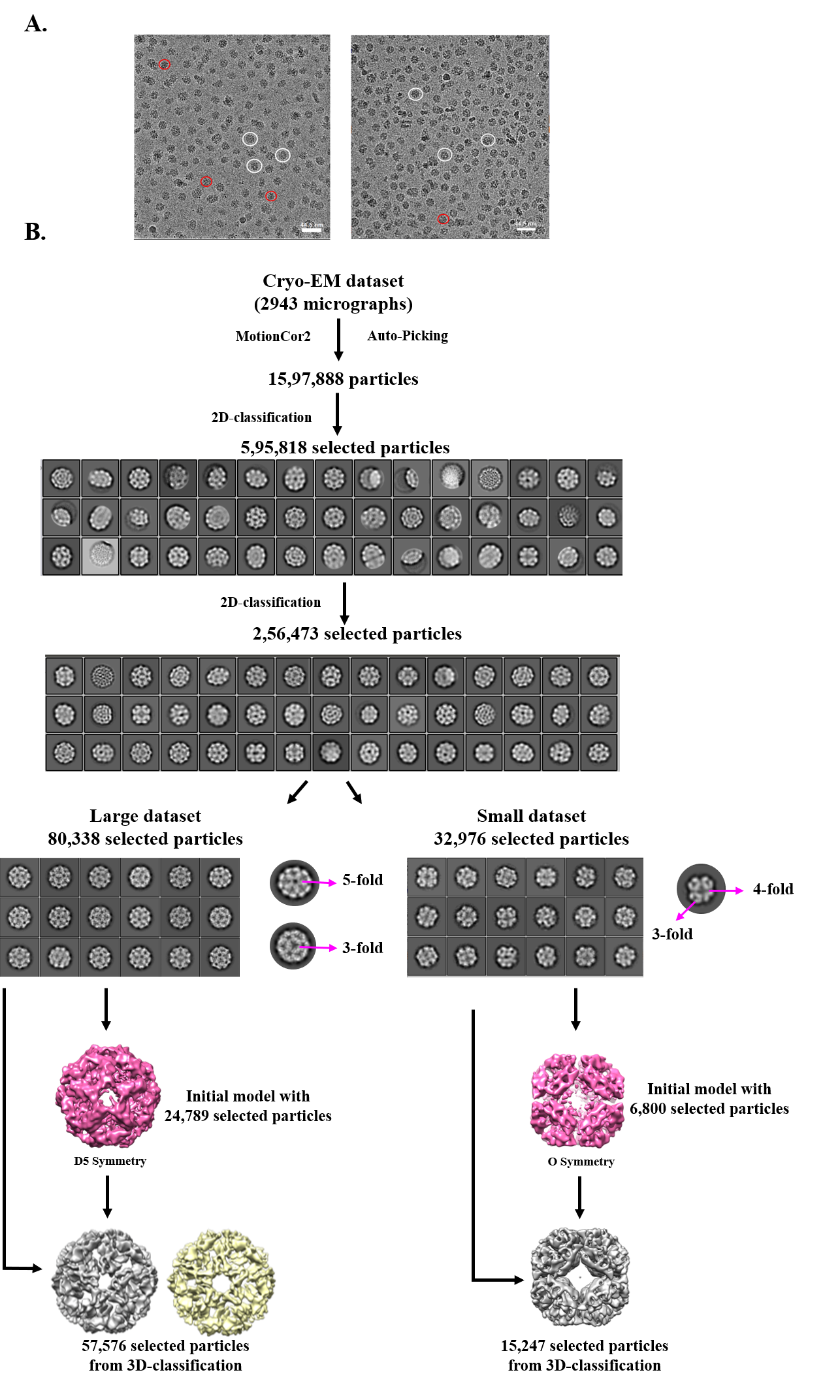


**Fig S5.** Cryo-EM analysis of SM2-FL (**A**) Micrographs (Scale bar, 44.5 nm). Two different oligomers are highlighted: the larger populations in white circles and the smaller populations in red circles, respectively. (**B**) Flowchart of the data processing by *RELION 3.0*. The stepwise 2D-classification, the separation of the “large” and “small” particle populations according to size and symmetry and various stages leading to final 3D image reconstruction are shown.


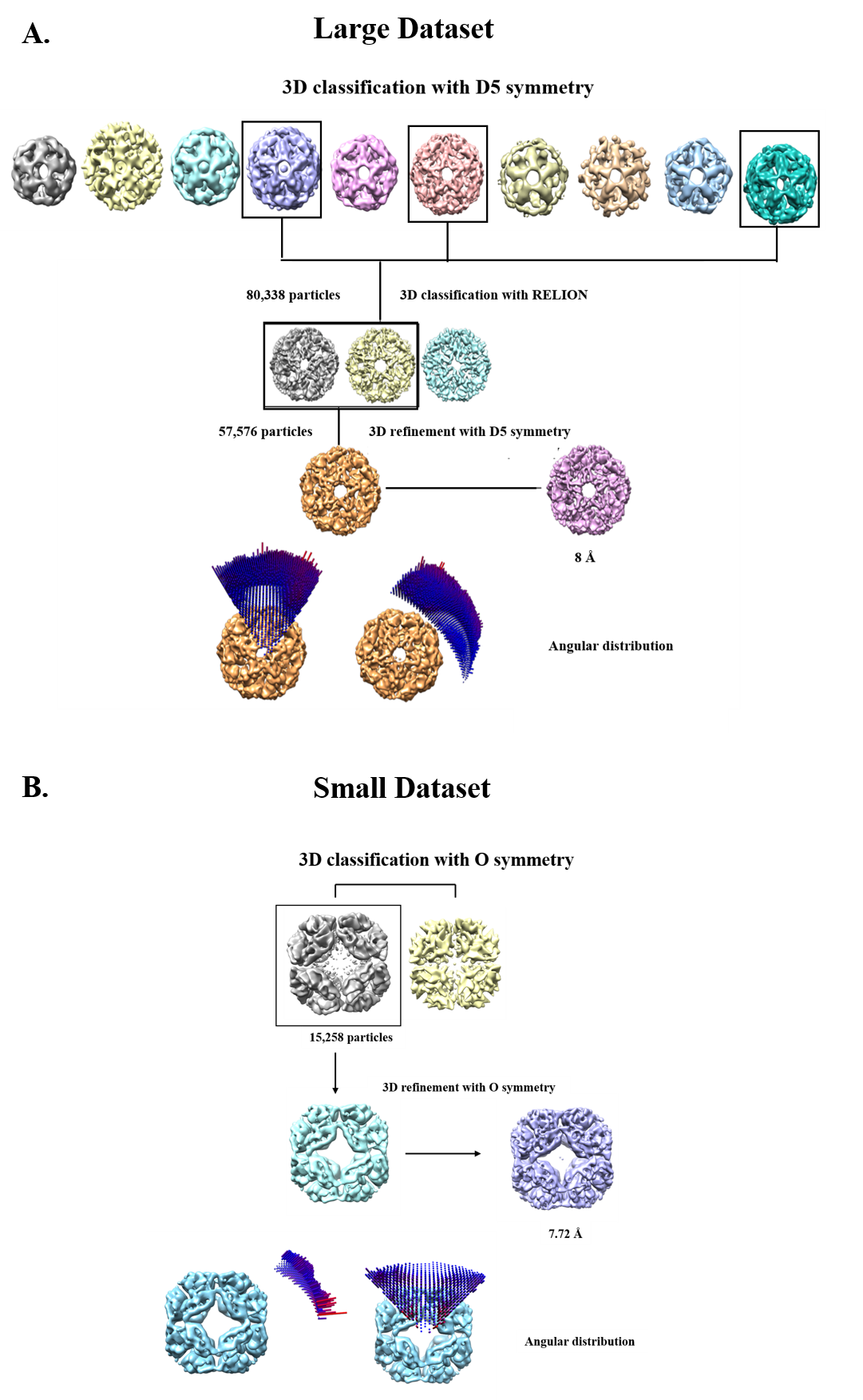


**Fig S6. A.** Processing of cryo-EM data of SM2-FL (**A**) “large dataset” with D5 symmetry and (**B**) the “small dataset” with O symmetry. The angular distribution shows red long rods indicating highest probability of image acquisition of the particle in a particular orientation.


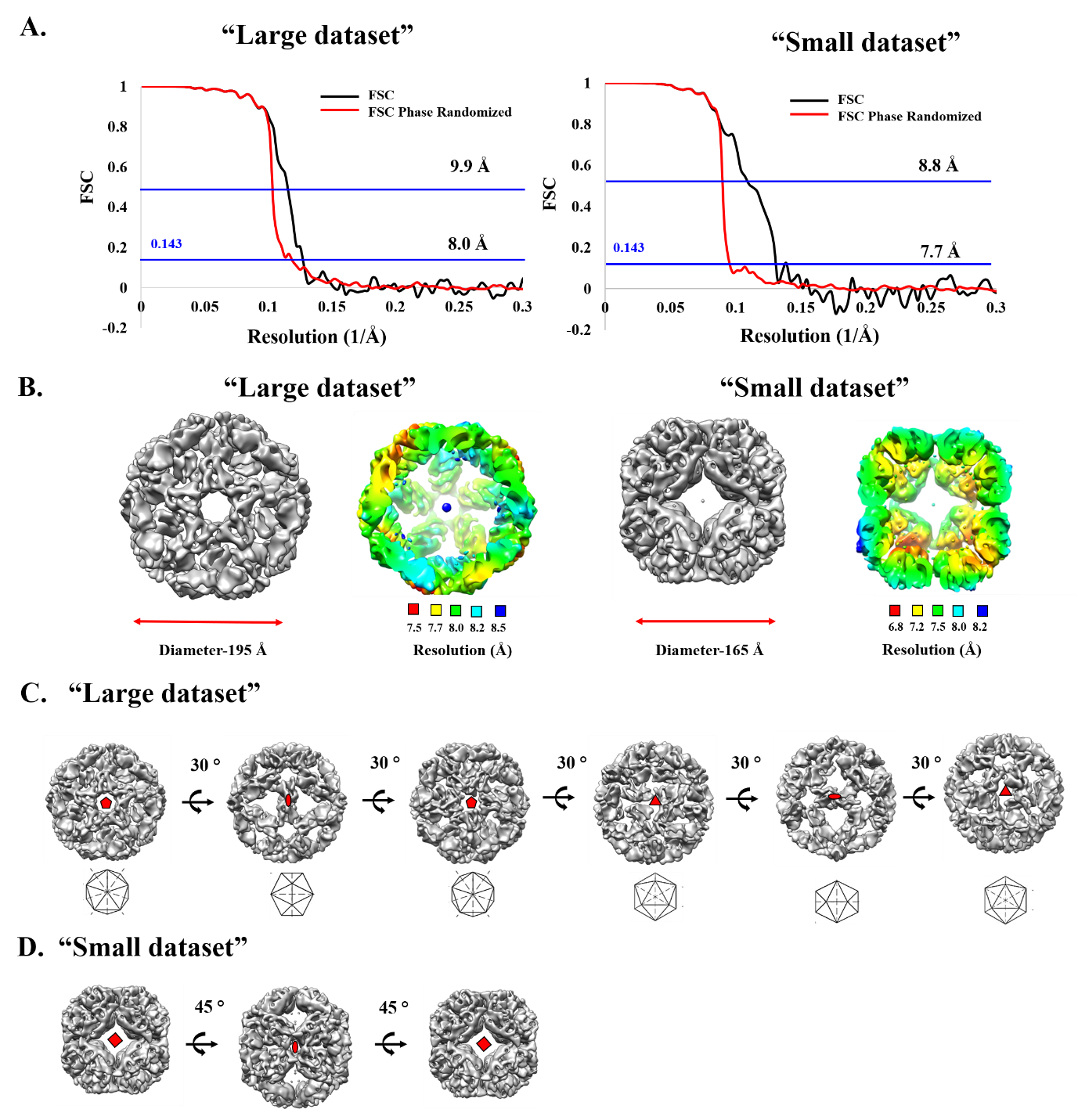


**Fig S7.** Analysis of the EM maps (**A**) FSC curve (**B**) Map diameter and Local resolution maps of “large” and “small” datasets. (**C, D**) View of EM maps at every 30° and 45° rotation of “large” and “small” dataset, respectively.


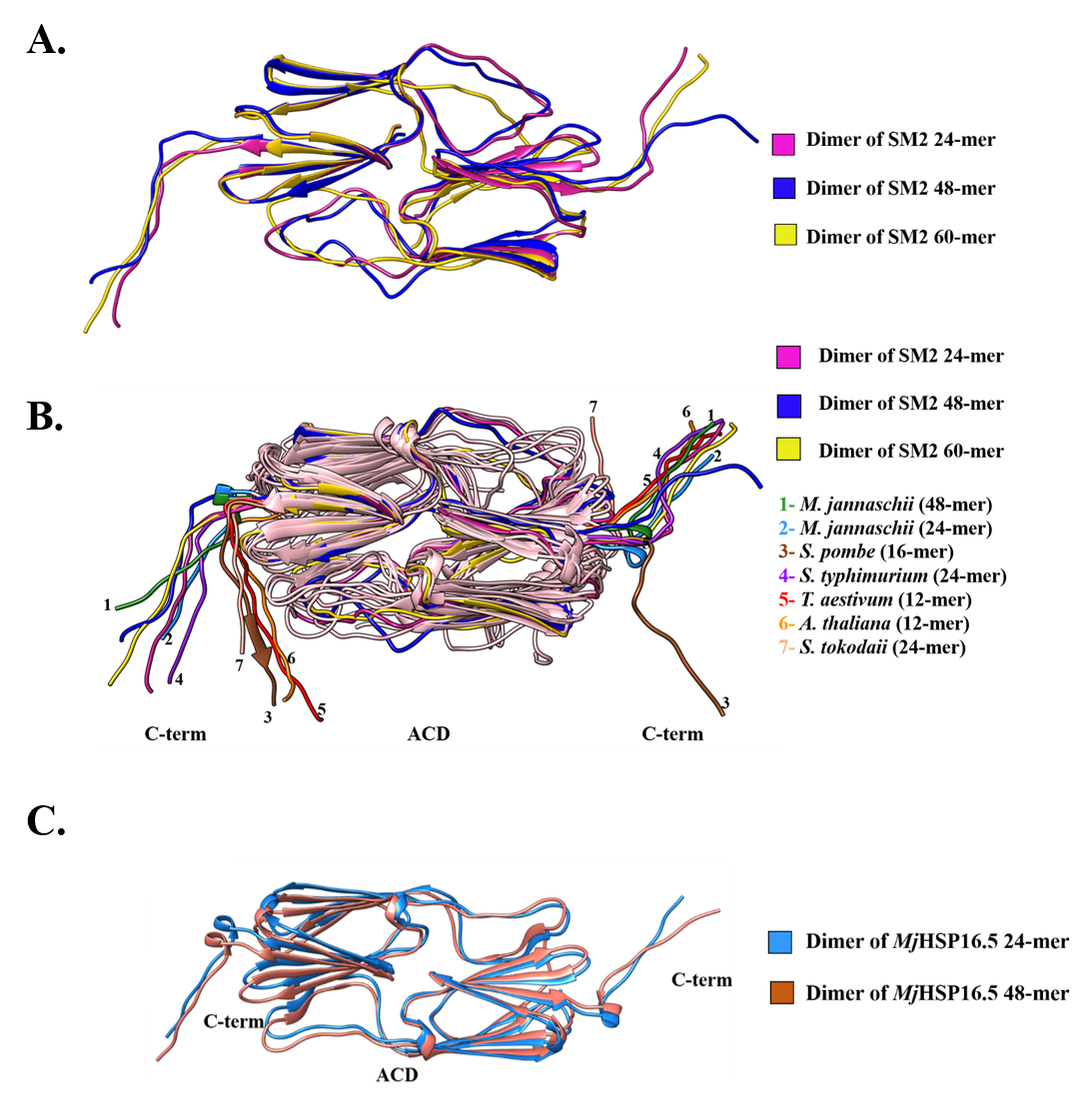


**Fig S8.** Superposition of dimers (**A**) Dimers of SM2 in different oligomers. (**B**) Superposition of SM2 dimers on dimers of other sHSP dimers (light pink) shows different orientations of the C-termini. (**C**) Superposition of the dimers of 24-mer (PDB code: 1SHS) and 48-mer (PDB code: 4ELD) of *Mj*HSP16.5.


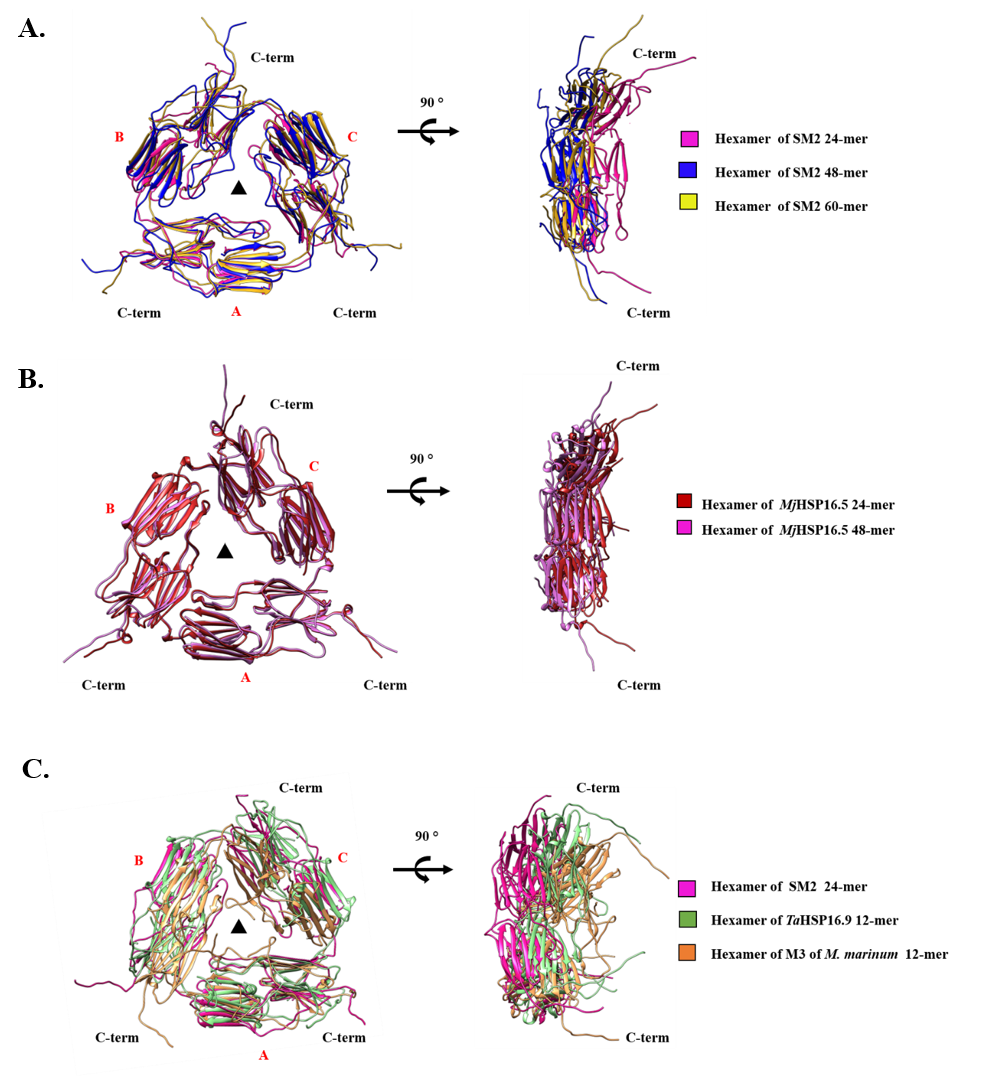


**Fig S9.** Structural superposition of the hexamers of (**A**) SM2 (**B**) *Mj*HSP16.5 from *M. jannaschii* 24mer (PDB code: 1SHS) and 48-mer (PDB code: 4ELD) and (**C**) *Ta*Hsp16.9 (PDB code: 1GME) and M3 from *M. marinum* (PDB code: 5ZS3) and SM2 24-mer. The dimer A of all the three hexamers were superposed.

**Reference**

1. Schindelin, J. *et al.* Fiji: An open-source platform for biological-image analysis. *Nature Methods*. **9**, 676–682 (2012).
